# Evolution of *Wolbachia* mutualism and reproductive parasitism: insight from two novel strains that co-infect cat fleas

**DOI:** 10.7717/peerj.10646

**Published:** 2020-12-17

**Authors:** Timothy P. Driscoll, Victoria I. Verhoeve, Cassia Brockway, Darin L. Shrewsberry, Mariah Plumer, Spiridon E. Sevdalis, John F. Beckmann, Laura M. Krueger, Kevin R. Macaluso, Abdu F. Azad, Joseph J. Gillespie

**Affiliations:** 1 Biology, West Virginia University, Morgantown, WV, USA; 2 Microbiology and Immunology, University of Maryland at Baltimore, Baltimore, MD, USA; 3 Entomology and Plant Pathology, Auburn University, Auburn, AL, USA; 4 Orange County Mosquito and Vector Control District, Garden Grove, CA, USA; 5 Microbiology and Immunology, University of South Alabama, Mobile, AL, USA

**Keywords:** *Wolbachia*, *Ctenocephalides felis*, Cat flea, Reproductive parasitism, Mutualism, Lateral gene transfer, Cytoplasmic incompatibility, Biotin operon

## Abstract

Wolbachiae are obligate intracellular bacteria that infect arthropods and certain nematodes. Usually maternally inherited, they may provision nutrients to (mutualism) or alter sexual biology of (reproductive parasitism) their invertebrate hosts. We report the assembly of closed genomes for two novel wolbachiae, *w*CfeT and *w*CfeJ, found co-infecting cat fleas (*Ctenocephalides felis*) of the Elward Laboratory colony (Soquel, CA, USA). *w*CfeT is basal to nearly all described *Wolbachia* supergroups, while *w*CfeJ is related to supergroups C, D and F. Both genomes contain laterally transferred genes that inform on the evolution of *Wolbachia* host associations. *w*CfeT carries the Biotin synthesis Operon of Obligate intracellular Microbes (BOOM); our analyses reveal five independent acquisitions of BOOM across the *Wolbachia* tree, indicating parallel evolution towards mutualism. Alternately, *w*CfeJ harbors a toxin-antidote operon analogous to the *w*Pip *cinAB* operon recently characterized as an inducer of cytoplasmic incompatibility (CI) in flies. *w*CfeJ *cinB* and three adjacent genes are collectively similar to large modular toxins encoded in CI-like operons of certain *Wolbachia* strains and *Rickettsia* species, signifying that CI toxins streamline by fission of large modular toxins. Remarkably, the *C*. *felis* genome itself contains two CI-like antidote genes, divergent from *w*CfeJ *cinA*, revealing episodic reproductive parasitism in cat fleas and evidencing mobility of CI loci independent of WO-phage. Additional screening revealed predominant co-infection (*w*CfeT/*w*CfeJ) amongst *C*. *felis* colonies, though fleas in wild populations mostly harbor *w*CfeT alone. Collectively, genomes of *w*CfeT, *w*CfeJ, and their cat flea host supply instances of lateral gene transfers that could drive transitions between parasitism and mutualism.

## Introduction

Wolbachiae (*Alphaproteobacteria*: Rickettsiales: Anaplasmataceae) comprise Gram-negative, obligate intracellular bacteria that infect over half the world’s described insect species as well as certain parasitic nematodes (Hilgenboecker et al., 2008). Unlike other notable rickettsial genera that contain human pathogens (e.g., *Rickettsia*, *Orientia*, *Neorickettsia*, *Anaplasma*, and *Ehrlichia*), wolbachiae do not infect vertebrates (Gillespie et al., 2012). A single species, *Wolbachia pipientis*, is formally recognized with numerous members designated as strains within 16 reported supergroups (Augustinos et al., 2011; Baldo & Werren, 2007; Bing et al., 2014; Glowska et al., 2015; Haegeman et al., 2009; Lo et al., 2002; Ros et al., 2009; Lefoulon et al., 2020). Genomic divergence indicates further species names are warranted (Chung et al., 2018), though increasing diversity and community consensus suggest caution regarding further *Wolbachia* classification at the species level (Lindsey et al., 2016; Ramírez-Puebla et al., 2015).

Like other obligate intracellular microbes, wolbachiae are metabolic parasites that complement a generally reduced metabolism with pilfering of host metabolites (Driscoll et al., 2017; Jiménez et al., 2019). Their ability to survive and flourish is also heavily influenced by the acquisition of key functions through lateral gene transfer (LGT). Several described *Wolbachia* strains demonstrate characteristics of limited mutualism with their invertebrate partner (Zug & Hammerstein, 2015; Hosokawa et al., 2010), through the synthesis and provisioning of riboflavin (Moriyama et al., 2015) and biotin (Ju et al., 2019; Nikoh et al., 2014). While riboflavin biosynthesis genes are highly conserved in wolbachiae (Newton & Rice, 2020), biotin biosynthesis genes are rare and likely originated via LGT with taxonomically divergent intracellular bacteria (Gillespie et al., 2012). Still other strains of *Wolbachia* exert varying degrees of reproductive parasitism (RP) on their insect host (Werren, Baldo & Clark, 2008), influencing host sexual reproduction via processes such as male-killing, feminization, parthenogenesis and cytoplasmic incompatibility (CI) (Hurst & Frost, 2015). *Wolbachia* genes underpinning CI and male killing have been characterized (Chen et al., 2019; Beckmann et al., 2019; LePage et al., 2017; Beckmann, Ronau & Hochstrasser, 2017; Beckmann & Fallon, 2013; Perlmutter et al., 2019) and occur predominantly in the eukaryotic association module (EAM) of *Wolbachia* prophage genomes (Bordenstein & Bordenstein, 2016). These genes highlight the role of LGT in providing wolbachiae with factors facilitating mutualism or RP, both of which are highly successful strategies for increasing infection frequency in invertebrate host populations.

Compared to reproductive parasites, *Wolbachia* mutualists appear to form more stable, long-term relationships with their hosts, as supported by *Wolbachia*-host codivergence in certain filarial nematodes (Bandi et al., 1998) and *Nomada* bees (Gerth & Bleidorn, 2017). In contrast to the stability of mutualists, relationships of reproductive parasites appear more ephemeral. RP can be a strong mechanism to increase infection frequency; for example, hosts of CI-inducing strains can rapidly replace uninfected members within populations (Turelli & Hoffmann, 1991; Schmidt et al., 2017). Despite this, CI is prone to neutralization through the evolution of host suppression (Hurst & Frost, 2015; Cooper et al., 2017; Turelli, 1994) and purifying selection on the host does not preserve CI (Turelli, 1994).

Recent work proposes that CI genes, or CI factors (cifs), have genetic life cycles (Martinez et al., 2020). Initially, cifs might colonize new hosts by LGT, pushing said host to high frequency in populations through CI. Ultimately, CI collapses as cifs become pseudogenized. Thus, cifs are selfish elements that persist in a manner similar to transposons (Martinez et al., 2020; Cooper et al., 2019). The occurrence of duplicate cif copies (i.e., encoding the same biochemical mechanism), as well as cif pseudogenes, in many *Wolbachia* strains supports this model (Gillespie et al., 2018). Cif degradation would lead to a weak CI background, fomenting favorable conditions for *Wolbachia* strains with more active cif repertoires to invade and outpace resident strains carrying failing cifs. An alternative to autonomous cif jumping would be recombinant WO-phages or smaller elements actually carrying CI operons. If dosage is important to CI, all these events might shift the strength of CI leading to new bidirectional incompatibilities.

Such LGT partly explains why *Wolbachia* estimated phylogenies are often discordant with those of their hosts (Cooper et al., 2019; Raychoudhury et al., 2009; Turelli et al., 2018). Horizontal transmissions of *Wolbachia* strains, which can occur through direct host interactions, environmental contacts (shared habitat, food sources, etc.) or predator/parasitoid delivery, also cause tree discordance (Pietri, DeBruhl & Sullivan, 2016) and contribute to episodic invasions, replacements, and exchange of cifs. Horizontal transmission is evident in cases where hosts are infected with multiple divergent *Wolbachia* strains (Raychoudhury et al., 2009; Turelli et al., 2018; Pietri, DeBruhl & Sullivan, 2016). In host populations that vary from single- vs double-infections, co-infection might reflect a transitory phase (i.e., one strain replacing another) or stable co-infection. We envision that competition could arise between co-infections if the distinct reproductive parasites carry too similar RP-inducing genes and battle for control of parallel reproductive mechanisms. Alternatively, perhaps the stability of co-infections could be supported in part by different mechanistic approaches to RP (e.g., *cin* vs *cid* loci) or dosage effects from multiple strains collaborating to instantiate a new crossing type. Furthermore, stable coinfections might increase robustness by partitioning distinct strains to serve within distinct host micro-niches (i.e., nutrient provisioning vs RP); each might increase fitness distinctly or alternatively parasitize distinctly. Such a case is in part witnessed in doubly-infected cochineals (*Dactylopius coccus*), wherein a Supergroup B strain (*w*DacB) carries an arsenal of RP-inducing genes but a Supergroup A strain (*w*DacA) carries none (Raychoudhury et al., 2009). Clearly, documenting more cases of *Wolbachia* co-infections putatively serving under contrasting host relationships will be helpful to understand mechanisms of replacement and stability in host populations.

We recently sequenced the genome of the cat flea (*Ctenocephalides felis*) and concurrently closed the genomes of two novel wolbachiae, *w*CfeT and *w*CfeJ, that co-infect fleas of the Elward Laboratory (Soquel, CA, USA; hereafter EL fleas) (Driscoll et al., 2020). Genome-based phylogeny estimation placed *w*CfeT on a basal divergent branch, while *w*CfeJ was found to subtend *Wolbachia* Supergroup C. Both genomes show evidence of past WO phage infection, with LGT hotspots indicating recent acquisition of factors predicted to either underpin mutualism (*w*CfeT) or mediate CI (*w*CfeJ). Additionally, we describe two loci within the *C. felis* genome itself that support repeated acquisitions of CI antidote genes by the host. Infection surveys of cat flea populations and colonies from across the US indicate extensive spread of *w*CfeT among wild populations, and a strong bias for *w*CfeT/*w*CfeJ co-infection in colonies, highlighting the complex ecological relationship that drives this system. Finally, we apply an informatics approach including phylogenomics, synteny analysis, and functional alignment to construct a comprehensive picture revealing the recurrent evolution of nutritional symbiosis and RP across the Rickettsiales.

## Materials and Methods

### Assembly and annotation of *w*CfeT and *w*CfeJ

Our recent report detailed (1) the assembly of *Wolbachia* reads generated from *C*. *felis* genome sequencing, (2) genome circularization and naming of two novel strains (*w*CfeT and *w*CfeJ), and (3) complete genome annotation (Driscoll et al., 2020). All relevant information pertaining to *w*CfeT and *w*CfeJ is available at NCBI under Bioproject PRJNA622233. Complete genome sequences are available in the NCBI RefSeq database at accession identifiers NZ_CP051156.1 (*w*CfeT) and NZ_CP051157.1 (*w*CfeJ).

### Wolbachiae phylogenomics

Protein sequences (*n* = 84,836) for 66 sequenced *Wolbachia* genomes plus five additional Anaplasmataceae (*Neorickettsia helminthoeca* str. Oregon, *Anaplasma centrale* Israel, *A. marginale* Florida, *Ehrlichia chaffeensis* Arkansas, and E. *ruminantium Gardel*) were either downloaded directly from NCBI (*n* = 48), retrieved as genome sequences from the NCBI Assembly database (*n* = 13), contributed via personal communication (*n* = 8; Michael Gerth, Oxford Brookes University), or sequenced here (*n* = 2) (Table S1). For genomes lacking functional annotations (*n* = 15), gene models were predicted using the RAST v2.0 server (*n* = 12) or GeneMarkS-2 v1.10_1.07 (*n* = 3; (Lomsadze et al., 2018)). Ortholog groups (*n* = 3,038) were subsequently constructed using FastOrtho, an in-house version of OrthoMCL (Li, Stoeckert & Roos, 2003), using previously established criteria (i.e., an expect threshold of 0.01, percent identity threshold of 30%, and percent match length threshold of 50% for ortholog inclusion (Driscoll et al., 2017, 2020; Smith et al., 2015; Hagen et al., 2018)). A subset of single-copy families (*n* = 12) conserved across at least 60 of the 66 genomes were independently aligned with MUSCLE v3.8.31 (Edgar, 2004) using default parameters. Regions of poor alignment were masked with trimal v1.4.rev15 (Capella-Gutiérrez, Silla-Martínez & Gabaldón, 2009) using the “automated1” option. All modified alignments were concatenated into a single data set (2,803 positions) for phylogeny estimation using RAxML v8.2.4 (Stamatakis, 2014). The gamma model of rate heterogeneity and estimation of the proportion of invariant sites were used. Branch support was assessed with 1,000 pseudo-replications. Final ML optimization likelihood was −52350.085098.

### BOOM characterization

#### Phylogeny estimation

Genomes (*n* = 93) containing at least four of the six biotin synthesis genes were identified using Blastp searches against the NCBI nr database (*e*-value < 0.01; accessed 9 January 2020). Protein sequences for these loci were downloaded from NCBI, aligned with MUSCLE v3.8.31 (Edgar, 2004) using default parameters, and regions of poor alignment masked with trimal v1.4.rev15 (Capella-Gutiérrez, Silla-Martínez & Gabaldón, 2009) using the “automated1” option. All modified alignments were concatenated into a single data set (1,457 positions) for phylogeny estimation using RAxML v8.2.4 (Stamatakis, 2014), under the gamma model of rate heterogeneity and estimation of the proportion of invariant sites. Branch support was assessed with 1,000 pseudo-replications. Final ML optimization likelihood was −127140.955066. See Table S2 for NCBI protein accession IDs for all sequences included in this analysis.

#### Gene neighborhood analysis of wCfeT BOOM

Genes in a 25-gene window around the *w*CfeT BOOM cluster were used to query (Blastp) the NCBI nr database (accessed 9 January 2020). The top 50 matches to each sequence (*e*-value < 1, BLOSUM45 matrix) were binned by taxonomic assignment as “Wolbachia”, “other Rickettsiales”, or “other (non-Rickettsiales) taxa.” Other (non-Rickettsiales) taxa were further binned by major taxonomic group to assess the boundaries and diversity of the *w*CfeT BOOM MGE.

#### Synteny analysis of biotin genes in wolbachiae

Rickettsiales genomes with at least 5 of the 6 biotin synthesis genes (*n* = 28) were downloaded from NCBI and ortholog groups (*n* = 2,527) constructed with FastOrtho, an in-house version of OrthoMCL (Li, Stoeckert & Roos, 2003), using an expect threshold of 0.01, percent identity threshold of 30%, and percent match length threshold of 50% for ortholog inclusion. OGs containing biotin synthesis genes were identified using *Rickettsia buchneri* str. Wikel sequences as seeds. Genome locations for all genes were retrieved from NCBI in gene file format (gff) and used to order the orthologs in each genome.

#### Phylogeny estimation of the EamA protein of wCfeT

Taxa were selected from Blastp searches against the NCBI Rickettsiales and nr (excluding Rickettsiales) databases using the *w*CfeT EamA protein (WP_168464633.1) as a query. Proteins were aligned with MUSCLE v3.8.31 (Edgar, 2004) using default parameters, with regions of poor alignment masked using Gblocks (Talavera & Castresana, 2007). Phylogeny was estimated with the WAG substitution model using RAxML v8.2.4 (Stamatakis, 2014) under the gamma model of rate heterogeneity and estimation of the proportion of invariant sites.

### CI gene characterization

#### Wolbachiae

*w*CfeJ CinA and CinB were compared to other RP-inducing TA operons following our previous approach (Gillespie et al., 2018). Another novel CndAB operon was identified on a small scaffold (NCBI Nucleotide ID CABPRJ010001055.1) in the aphid *Cinara cedri*, which has an associated *Wolbachia* symbiont, *w*Ced (Gómez-Valero et al., 2004), and was also characterized in a similar manner. Briefly, EMBL’s Simple Modular Architecture Research Tool (SMART) (Letunic & Bork, 2017) and /or the Protein Homology/analogY Recognition Engine V 2.0 (Phyre2) (Kelley & Sternberg, 2009) were used to predict the following domains: OTU cysteine protease (Pfam OTU, PF02338) (Makarova, Aravind & Koonin, 2000), endoMS-like (Nakae et al., 2016) and NucS-like (Ren et al., 2009) PD-(D/E)XK nuclease (Pfam PDDEXK_1, PF12705) (Kinch et al., 2005), CE clan protease (Pfam Peptidase_C1, PF00112) (Pruneda et al., 2016), Latrotoxin-CTD (Zhang et al., 2012), and ankyrin repeats. Individual protein schemas were generated using Illustrator of Biological Sequences (Liu et al., 2015) with manual adjustment.

#### C. felis

A *cidA*-like gene was identified in the *Ctenocephalides felis* genome during initial genome decontamination (Driscoll et al., 2020). Briefly, a contig composed of a mosaic of flea- and *Wolbachia*-like sequence was identified by our comparative BLAST-based pipeline and flagged for manual inspection. Gene models on this contig were predicted using the RAST v2.0 server. The resulting proteins were used to create a custom Blast database that was queried using Blastp with CinAB sequences from *Wolbachia* endosymbiont of *Culex quinquefasciatus* Pel (CAQ54390, CAQ54391), and CidAB sequences *Wolbachia pipientis* wAlbB (CCE77512, CCE77513). The contig was subsequently confirmed as belonging to the largest scaffold (NW_020538040) in the *C. felis* assembly. A second, *cinA*-like gene was identified by querying all CDS in the published *C. felis* assembly (NCBI accession ASM342690v1) with the same set of *Wolbachia* CinAB and CidAB sequences.

### Screening colonies and wild populations of cat fleas for *w*CfeT and *w*CfeJ

Upon receipt from collaborators, cat fleas were stored at −20 °C in EtOH until processing for DNA extraction and qPCR. Fleas were sexed under a stereomicroscope and surface sterilized for 5 min with 1% bleach solution, followed by 5 min with 70% EtOH, and finally with three washes using molecular grade water. Individual fleas were flash-frozen in liquid N_2_ and ground to powder with sterile pestle in a 1.5 ml microcentrifuge tube. DNA was extracted from dissected tissues using the Zymo Quick-DNA Miniprep plus kit following the solid tissue protocol (Zymo Research; Irvine, CA, USA). The presence of *w*CfeT, *w*CfeJ, and *C. felis* DNA was determined via qPCR using the following primer sets: Cfe#18S|179, 5′-TGCTCACCGTTTGACTTGG-3′ and 5′-GTTTCTCAGGCTCCCTCTCC-3′ (Reif et al., 2008); *w*CfeT#ApaG|75: 5′-GCCGTCACTGGCAGGTAATA-3′ and 5′-GCTGTTCTCCAATAACGCCA-3′, *w*CfeJ#CinA|76, 5′-AGCAACACCAACATGCGATT-3′ and 5′-GAACCCCAGAGTTGGAAGGG-3′. Each primer set amplicon was sequenced before use to ensure specificity. qPCR was performed using a QuantStudio 3 with the PowerUp SYBR green mastermix in a 20 μl reaction volume containing 2 μl of sample DNA with 400 nM of each primer. Cycling parameters were consistent with the “fast cycling mode” including a 2 min cycle at 50 °C, a 2 min cycle at 95 °C, 40 cycles with 3 s at 95 °C and 30 s at 60 °C followed by a melt curve analysis. Standard curves were generated to quantify the number of DNA copies present in each reaction. Results were expressed as the ratio of *Wolbachia* amplicons to *C. felis* 18S rDNA copies to normalize for varying efficiency of DNA extractions. Fleas were considered positive for wolbachiae if the flea 18S rDNA Ct value is 30 or lower and the *Wolbachia* Ct value is 37 or lower.

### Determining *w*CfeT and *w*CfeJ localization in *C*. *felis* tissues

Elward Laboratories (EL, Soquel, CA, USA) cat fleas stored at −80 °C were used to assay tissue localization of *w*CfeT and *w*CfeJ. Ovaries and midguts of individual female fleas were dissected under a stereomicroscope. DNA extraction and qPCR were performed as described above.

## Results and Discussion

Fleas are understudied vectors of several human and animal diseases (Rust, 2017). While wolbachiae (mostly unnamed strains) have been detected in many flea species (Gorham, Fang & Durden, 2003; Dittmar & Whiting, 2004; Lawrence et al., 2015), it is pressing from a biocontrol perspective to know if these bacteria affect the ability of fleas to vector pathogens. The cat flea transmits pathogenic bacteria such as *Rickettsia typhi* (murine typhus), *R*. *felis* (murine typhus-like illness), *Bartonella* spp. (e.g., cat-scratch disease and various other bartonelloses), and to a lesser extent the plague agent *Yersinia pestis* (Glickman et al., 2006; Nogueras et al., 2013; Angelakis et al., 2016; Leulmi et al., 2014). Cat fleas are also intermediate hosts of the tapeworm *Dipylidium caninum* and the filarial nematode *Acanthocheilonema reconditum*, both of which can infect humans (Fourie et al., 2012; Brianti et al., 2012; Traversa, 2013). Other organisms observed in *C*. *felis* include undescribed Baculoviridae, amebae, trypanosomatids, cephaline gregarines (Apicomplexa), and microsporidia (Beard, Butler & Hall, 1990). However, nearly all epidemiological studies have focused on determining the frequency and distribution of only certain pathogens (e.g., *Bartonella* spp., *R*. *typhi*, *R*. *felis*), with few documenting wolbachiae co-occurrence with these pathogens (Tay, 2013; Oteo et al., 2014).

### Identification of novel *Wolbachia* parasites

Recently, we assembled and annotated closed genomes from two divergent *Wolbachia* strains, named *w*CfeT and *w*CfeJ, that were captured during genome sequencing of the cat flea (EL Colony) (Driscoll et al., 2020). The 16S rDNA sequences of these two strains are identical to those previously identified in another cat flea colony (Louisiana State University) (Pornwiroon et al., 2007; Sunyakumthorn et al., 2008; Gillespie et al., 2015), which historically has been replenished with EL colony fleas. *w*CfeT and *w*CfeJ are broadly similar to other closed *Wolbachia* genomes in terms of length, GC content, number of protein coding genes, and coding density (Table 1), although it is interesting to note that *w*CfeT exhibits one of the highest pseudogene counts (second only to *w*Cle) while *w*CfeJ boasts one of the lowest (second only to *w*Ov). Coupled with other recently sequenced genomes for non-Supergroup A and B strains (e.g., *w*Fol and *w*VulC), these cat flea-associated wolbachiae blur the lines demarcating genomic traits previously used to distinguish basal and sister *Wolbachia* lineages from those of Supergroups A and B strains (Lefoulon et al., 2020), particularly regarding genome size and mobile element composition (Lefoulon et al., 2020).

**Table 1:** Summary statistics of selected *Wolbachia* genomes.

Taxon	RefSeq ID	SG*	Length (nt)	GC (%)	Protein coding genes	Pseudogenes	Mean gene length (nt)	Coding density (coding/total nt)
*w*Fol	NZ_CP015510.2	E	1801626	34.4	1537	85	976	0.880
*w*CfeT	NZ_CP051156.1	?	1495538	35.2	1424	201	890	0.727
*w*Ov	NZ_HG810405.1	C	960618	32.1	650	45	967	0.700
*w*Oo	NC_018267.1	C	957990	32.1	639	73	968	0.719
*w*CfeJ	NZ_CP051157.1	?	1201647	35.6	1116	51	917	0.813
*w*Cle	NZ_AP013028.1	F	1250060	36.3	1012	210	818	0.829
*w*Bm	NZ_CP034333.1	D	1080064	34.2	803	178	868	0.789
*w*Bm	NC_006833.1	D	1080084	34.2	839	167	854	0.795
*w*Mau	NZ_CP034334.1	B	1273527	34.0	1045	136	944	0.876
*w*Mau	NZ_CP034335.1	B	1273530	34.0	1047	131	946	0.876
*w*No	NC_021084.1	B	1301823	34.0	1066	133	952	0.878
*w*AlbB FL2016	NZ_CP041923.1	B	1482279	34.5	1190	195	917	0.858
*w*AlbB HN2016	NZ_CP041924.1	B	1483853	34.4	1200	187	917	0.858
*w*AlbB	NZ_CP031221.1	B	1484007	34.4	1197	191	916	0.858
*w*Meg	NZ_CP021120.1	B	1376868	34.0	1136	125	948	0.869
*w*Ha	NC_021089.1	A	1295804	35.1	1123	104	899	0.852
*w*CauA	NZ_CP041215.1	A	1449344	35.0	1260	134	861	0.860
*w*Ana	NZ_CP042904.1	A	1401460	35.2	1218	107	893	0.857
*w*Ri	NC_012416.1	A	1445873	35.2	1245	122	892	0.859
*w*Au	NZ_LK055284.1	A	1268461	35.2	1099	125	878	0.855
*w*Mel	NC_002978.6	A	1267782	35.2	1116	129	863	0.857
*w*Mel I23	NZ_CP042444.1	A	1269137	35.2	1119	128	862	0.857
*w*Mel N25	NZ_CP042446.1	A	1267781	35.2	1120	125	863	0.857
*w*Mel ZH26	NZ_CP042445.1	A	1267436	35.2	1117	125	865	0.857
*w*Irr	NZ_CP037426.1	A	1352354	35.3	1232	115	827	0.855

**Note:**

* Supergroup designations for *w*CfeT and *w*CfeJ remain unclear.

**Figure 1:**
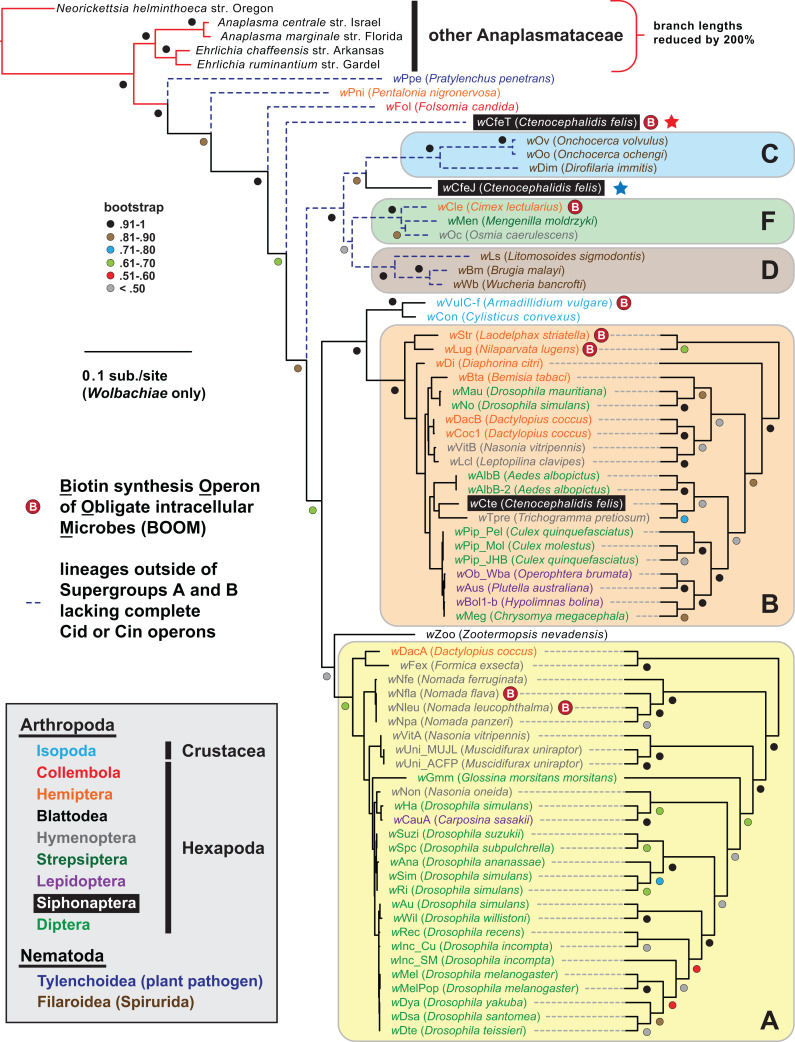
*Wolbachia* genome-based phylogeny estimation. *Wolbachia* supergroups are within colored ellipses. *Ctenocephalides felis*-associated Wolbachiae are within black boxes, with red (*w*CfeT) and blue (*w*CfeJ) stars depicting the two novel *Wolbachia* parasites infecting *C*. *felis*. Gray inset: color scheme for nematode and arthropod hosts. Phylogeny was estimated for 66 *Wolbachia* and five outgroup genomes (see “Materials and Methods” for more details). Branch support was assessed with 1,000 pseudoreplications. Final ML optimization likelihood was −52350.085098.

Prior reports have indicated the presence of multiple *Wolbachia* “types” infecting *C*. *felis*, with phylogenies inferred from partial gene sequences placing these unnamed *Wolbachia* strains in supergroups B or F, or in basal lineages (Gorham, Fang & Durden, 2003; Dittmar & Whiting, 2004; Casiraghi et al., 2005). Our robust genome-based phylogeny estimation reveals that both *w*CfeT and *w*CfeJ are divergent from Supergroup A and B wolbachiae (Fig. 1; Table S1). *w*CfeT is similar to undescribed *C*. *felis*-associated strains that branch basally to most other *Wolbachia* lineages (Gorham, Fang & Durden, 2003; González-Álvarez et al., 2017; Vasconcelos et al., 2018), while *w*CfeJ is similar to undescribed *C*. *felis*-associated strains closely related to *Wolbachia* supergroups C, D and F (Gorham, Fang & Durden, 2003; Casiraghi et al., 2005). The substantial divergence of *w*CfeT and *w*CfeJ from a *Wolbachia* supergroup B strain associated with *C*. *felis* from Germany (*w*Cte) indicates a diversity of wolbachiae that are capable of infecting cat fleas. Importantly, the nature of host-microbe relationships for any of these *C*. *felis*-associated wolbachiae is unknown.

### *w*CfeT is equipped with biotin synthesis genes

**Figure 2:**
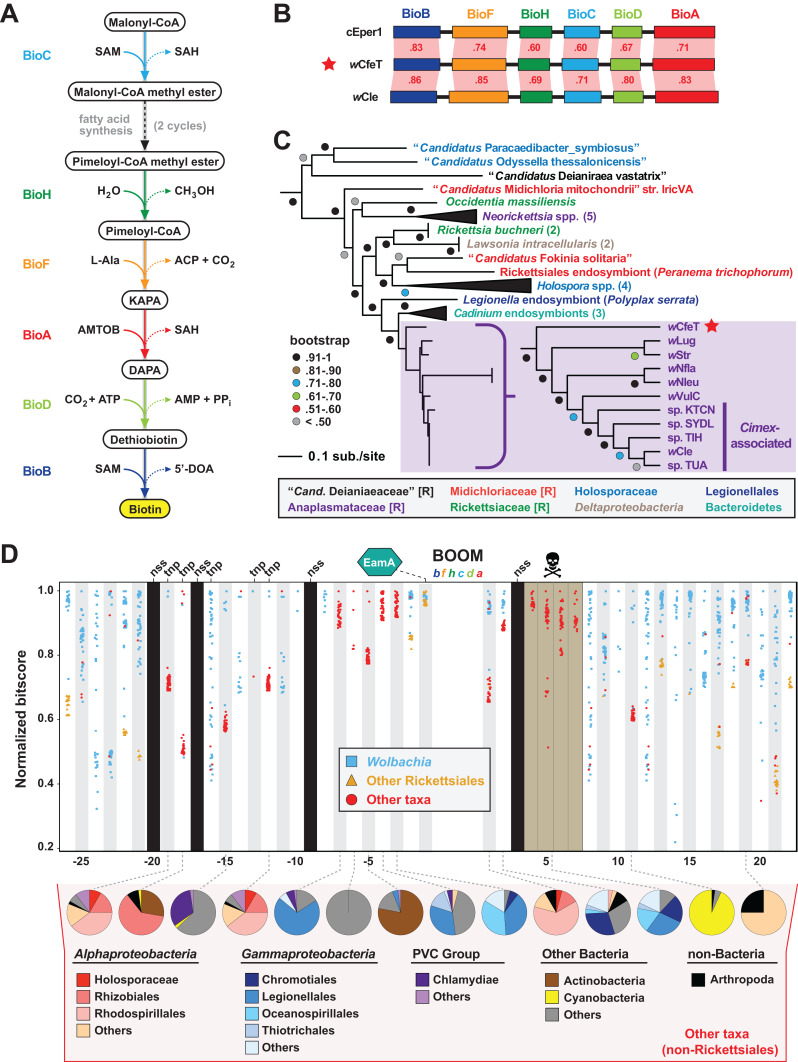
*w*CfeT carries the Biotin synthesis Operon of Obligate intracellular Microbes (BOOM). (A) Metabolism of biotin from malonyl-CoA (Lin & Cronan, 2011). (B) Comparisons of *w*CfeT BOOM to equivalent loci in *Cardinium* endosymbiont of *Encarsia pergandiella* (cEper1, CCM10336–CCM10341) and *Wolbachia* endosymbiont of *Cimex lectularius* (*w*Cle, BAP00143–BAP00148). Red shading and numbers indicate % identity across pairwise protein alignments (Blastp). Gene colors correspond to enzymatic steps depicted in panel A, with all proteins drawn to scale (as a reference, *w*CfeT BioB is 316 aa). (C) Phylogeny of BOOM and other *bio* gene sets from diverse bacteria estimated from the concatenation of six *bio* enzymes (BioC, BioH, BioF, BioA, BioD, and BioB); see Table S2 for all sequence information, and Fig. S1A for complete tree estimated under Maximum Likelihood. In taxon color scheme, [R] denotes Rickettsiales, with Holosporaceae as a revised family of Rhodospirillales (Muñoz-Gómez et al., 2019). (D) Analysis of genes flanking *w*CfeT BOOM. See Table S3 for comparison with other BOOM-containing wolbachiae. Graph depicts top 50 Blastp subjects (*e*-value < 1, BLOSUM45 matrix) using the *w*CfeT proteins as queries against the NCBI nr database (January 9, 2020). Colors distinguish three taxonomic groups (see inset). Black columns: no significant similarity (nss) in the NCBI nr database. Tnp, predicted transposase. Brown, pseudogenization. See Fig. S1B for EamA phylogeny estimation. Pie charts across the bottom delineate taxonomic diversity of hits to “Other taxa,” for *w*CfeT proteins with significant Blastp matches outside of the Rickettsiales.

The enzymatic steps for bacterial biosynthesis of biotin from malonyl-CoA are largely conserved (Lin & Cronan, 2011) and usually involve six *bio* enzymes and the fatty acid biosynthesis machinery (Lin, Hanson & Cronan, 2010) (Fig. 2A). We previously identified the first plasmid-borne *bio* gene operon, which occurs on plasmid pREIS2 of *Rickettsia buchneri*, an endosymbiont of the black-legged tick (Gillespie et al., 2012). We observed a rare gene order for the pREIS2 *bio* operon shared only with *bio* operons encoded on the chromosomes of obligate intracellular pathogens *Neorickettsia* spp. and *Lawsonia intracellularis* (*Deltaproteobacteria*). These unique *bio* operons formed a clade in estimated phylogenies, indicating LGT of the operon across divergent intracellular species. Subsequent studies, using our same dataset for phylogeny estimation, discovered this *bio* operon in certain *Wolbachia* (Nikoh et al., 2014; Gerth & Bleidorn, 2017; Balvín et al., 2018), *Cardinium* (Penz et al., 2012; Zeng et al., 2018) and *Legionella* (Ríhová et al., 2017) species. We refer hereafter to this intriguing LGT of *bio* genes as the Biotin synthesis Operon of Obligate intracellular Microbes (BOOM).

*w*CfeT carries a complete BOOM with greater similarity to *Wolbachia* BOOM than those of other bacteria (Fig. 2B). Phylogeny estimation of BOOM and other diverse *bio* gene sets indicates *w*CfeT BOOM is basal to other *Wolbachia* BOOM (Fig. 2C; Fig. S1). However, as the divergence pattern for other *Wolbachia* BOOM is discordant with genome-based phylogeny (Fig. 1), it is clear that multiple independent BOOM acquisitions have occurred in wolbachiae irrespective of lineage divergences. This is supported by a previous study positing recent BOOM acquisition in *w*Nfla and *w*Nleu (Gerth & Bleidorn, 2017) and a current report that detected partial BOOM loci in *w*Apol, a Supergroup S *Wolbachia* strain infecting pseudoscorpions (Lefoulon et al., 2020). The nature of BOOM flanking regions in the genomes of *w*CfeT, *w*VulC, *w*Cle, *w*Str, and *w*Lug, which collectively lack synteny and are riddled with transposases, recombinases and phage-related elements (Table S3), also attests to LGT as the origin for BOOM in these genomes. Furthermore, analysis of *w*CfeT BOOM flanking genes reveals hotspots for additional LGT (Fig. 2D), exemplified by an S-adenosylmethionine importer (EamA) gene (Fig. S1B) that is a signature among many diverse obligate intracellular bacteria (Gillespie et al., 2012; Klinges et al., 2019; Haferkamp et al., 2013; Driscoll et al., 2013).

As some BOOM-containing *Wolbachia* strains have been shown to provide biotin to their insect hosts (Ju et al., 2019; Nikoh et al., 2014), we posit that *w*CfeT has established an obligate mutualism with *C*. *felis* mediated by biotin-provisioning. Genes involved in CI (and putatively in MK) have been predominantly found in the EAM; the absence of such genes in *w*CfeT’s WO prophage (Fig. S2) provides no evidence that *w*CfeT can induce these two forms of RP, implying that its maintenance may indeed rely on nutritional supplementation to the cat flea. *Wolbachia* strains associated with bedbug (*w*Cle), planthopper (*w*Str, *w*Lug), and termites (*w*Ctub) with evidence for BOOM (Hellemans et al., 2019) reside in bacteriocytes of their insect hosts, structures associated with nutritional symbiosis in many arthropods (Douglas, 2015). Interestingly, *C*. *felis* is not known to contain bacteriocytes, and the ability of *w*CfeT to synthesize and supplement biotin to cat fleas awaits experimentation.

### *w*CfeJ lends insight on the evolution of reproductive parasitism

**Figure 3:**
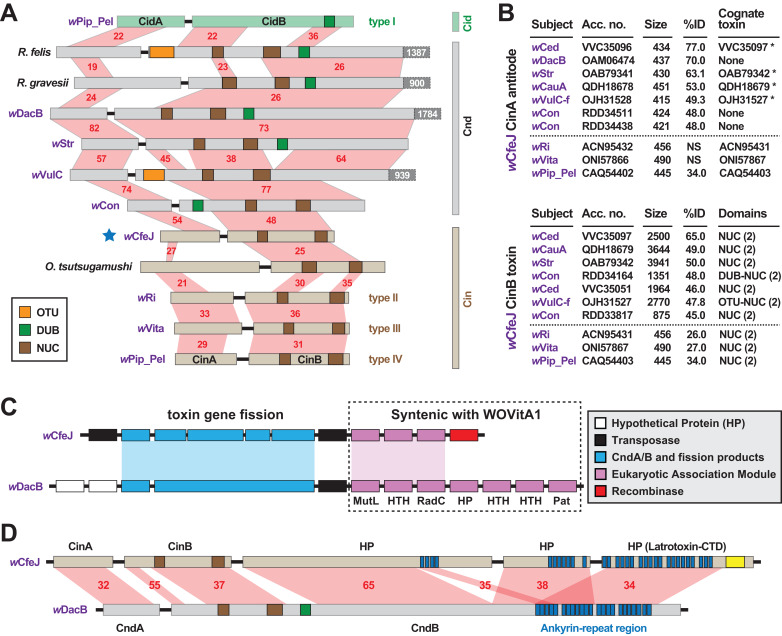
*w*CfeJ lends insight on the evolution of reproductive parasitism. (A) Comparison of known and predicted RP-inducing toxin-antidote operons of diverse rickettsial species. Light green: CidA/B of wPip_Pel (CAQ54390, CAQ54391); gray: CndA/B of pLbAR_36/38 (KHO02168, KHO02170), *Rickettsia gravesii* (WP_017442886, WP_024547315), *w*DacB (OAM06111, OAM06112), *w*Str (OAB79364, OAB79365), *w*VulC (OJH31528, OJH31527), *w*Con (RDD34163, RDD34164); light brown, CinA/B of *w*CfeJ (blue star, WP_168456002 & WP_168456001), *Orientia tsutsugamushi* (CAM80637, CAM80639), *w*Ri (ACN95432, ACN95431), *w*Vita (ONI57866, ONI57867), and wPip_Pel (CAQ54402, CAQ54403). Type I-IV CI operons of *Wolbachia* parasites (LePage et al., 2017) are distinguished. Protein domains as follows: green, CE clan protease; brown, PD-(D/E)XK nuclease; orange, OTU cysteine protease. Red shading and numbers indicate % identity across pairwise alignments. Proteins are drawn to scale, with additional C-terminal sequence for some CndB toxins depicted in dark gray boxes. (B) Sequence similarity profiles for *w*CfeJ’s CinA antidote (top) and CinA toxin (bottom). Subjects are from a Blastp search against the NCBI “Wolbachia” database. Size, amino acids. Antidotes with cognate toxins (operon partners) also recovered in searches are noted with asterisks. Top seven hits are shown, with matches (where significant) to proteins of types II-IV TA operons shown below dashed lines. Toxin domains are from our prior report or predicted newly here using the EMBL’s Simple Modular Architecture Research Tool (SMART) (Letunic & Bork, 2017). Only NUC and DUB domains are listed, though larger toxins contain many other predicted domains. (C) Comparison of *w*CfeJ and *w*DacB degraded WO prophage genomes captures streamlining of a CI-like TA operon from a Cnd operon. WO prophage coordinates: *w*CfeJ, WP_168455994–WP_168456003; *w*DacB, OAM06111–OAM06118 (LSYY01000098). Schema follows the description of the WOVitA prophage (Bordenstein & Bordenstein, 2016). (D) *w*CfeJ’s CinB-like toxin evolved from gene fission of a larger CndB-like toxin. NCBI protein accession numbers: *w*CfeJ (WP_168455999, WP_168456000, and WP_168456001); *w*DacB (OAM06111, OAM06112). Red shading and numbers indicate % identity across pairwise alignments. Proteins are drawn to scale; as a reference, *w*DacB CndB is 3707 aa.

*w*CfeJ carries a putative toxin-antidote (TA) operon that is architecturally similar to the *w*Pip_Pel CinA/B TA operon (Fig. 3A), which was previously characterized as a CI inducer in flies (Chen et al., 2019). Containing at least three distinct variants (LePage et al., 2017; Lindsey et al., 2018), all CinB toxins harbor dual nuclease (NUC) domains in place of the deubiquitinase (DUB) domain of CidB, another CI inducer in flies (LePage et al., 2017; Beckmann, Ronau & Hochstrasser, 2017; Beckmann & Fallon, 2013). Chimeric NUC-DUB toxins, termed CndB toxins (Beckmann et al., 2019), also occur as operons (*cndAB*) in certain *Wolbachia* and *Rickettsia* genomes (Beckmann, Ronau & Hochstrasser, 2017; Gillespie et al., 2018). Although their functions remain unknown, these larger CndB toxins belong to an extraordinarily diverse array of toxins with highly modular architectures that occur in divergent intracellular bacteria (Gillespie et al., 2018). Curiously, despite a size similar to the CinB toxins, *w*CfeJ’s CinB toxin has much higher sequence identity to larger CndB and NUC-containing toxins found mostly in *Wolbachia* Supergroups A and B (Fig. 3B). A similar pattern was found for *w*CfeJ’s CinA antidote, indicating the genes were likely acquired as an operon.

In contrast to *w*CfeT, *w*CfeJ harbors a highly degraded WO prophage genome with only a partial EAM, the CinA/B operon, and several other genes encoding hypothetical proteins and transposases (Fig. 3C). This is reminiscent of the CI- and male killer-inducing *w*Rec strain, which lacks a functional WO phage yet possesses TA modules within remnant EAMs (Metcalf et al., 2014).

Synteny analysis with other *Wolbachia* phages revealed *w*CfeJ *cinB* and three downstream genes are colinear with a large (3707 aa) *w*DacB *cndB* toxin (Fig. 3D). We posit that gene fission of a large modular CndB toxin created the smaller CinB toxin. This interpretation is consistent with our prior hypothesis stating that more streamlined RP-inducing toxins (i.e., *cinB* and *cidB*) originate from larger modular toxins (i.e., *cndB* and others) that are widespread in the intracellular mobilome, particularly *Wolbachia* phage and *Rickettsia* plasmids and integrative and conjugative elements (ICEs) (Gillespie et al., 2018, 2015). We find the alternative hypothesis, that multiple gene fusions create larger modular toxins like CndB proteins, less parsimonious provided that RP toxins tend to degrade and pseudogenize rapidly in rickettsial genomes (Martinez et al., 2020; Gillespie et al., 2018).

*w*CfeJ is unique among described wolbachiae outside of Supergroups A and B by containing a CI-like TA operon. Some of the *Wolbachia* strains in closely related Supergroups C, F and D are considered mutualists with their nematode or arthropod hosts, supporting the absence of RP-inducing genes in the genomes of these strains. Still, other strains not included in our analysis, namely those belonging to Supergroups S (Lefoulon et al., 2020; Chafee et al., 2010), P (Glowska et al., 2015), and J (Lefoulon et al., 2016) may eventually be shown to carry RP-inducing genes, especially since some of these strains are not known as mutualists. *w*Fol, another basal *Wolbachia* lineage (Fig. 1), does carry a myriad of large modular candidate RP-inducing toxins but does not have *cin*, *cid* or *cnd* TA operons (Faddeeva-Vakhrusheva et al., 2017; Kampfraath et al., 2019). This indicates LGT of candidate RP-inducing genes occurs across the breadth of *Wolbachia* diversity. Collectively, we speculate that *w*CfeJ acquired a WO prophage, possibly from a Supergroup A or B *Wolbachia* strain that also infects cat fleas (e.g., *w*Cte), and that viral- or *gene*-level selection streamlined a *cndAB* locus into a *cinAB* operon (Gillespie et al., 2018). Given that the genomes of many *Wolbachia* reproductive parasites harbor diverse arrays of CinA/B-and CidA/B-like operons (Gillespie et al., 2018; Beckmann et al., 2019), we posit that *w*CfeJ’s CinA/B TA operon might function in CI or some other form of RP; however, further study is required to evaluate *w*CfeJ as a cat flea reproductive parasite. Furthermore, an intriguing conjecture is that functions of the larger modular toxins, exemplified by *w*DacB CndB, whatever they be, are likely the primal functions that gave rise to CI.

### Lateral transfer of *Wolbachia* CI-like antidotes to the cat flea genome

Our previous work identified CI-like toxin and antidote genes on scaffolds in several arthropod genome assemblies, although antidote genes were found more frequently possibly due to utility in suppressing microbial invaders implementing RP (Gillespie et al., 2018). Many of these genomes contain multiple divergent antidote genes, though none were found to harbor large-scale *Wolbachia* gene transfer that is known to occur in certain invertebrates (Leclercq et al., 2016; Hotopp et al., 2007; Aikawa et al., 2009; Kondo et al., 2002; Dhaygude et al., 2019; McNulty et al., 2010; Werren et al., 2010). In light of this observation, we originally speculated that CI-like genes are mobilized from wolbachiae to host through WO phage insertion into host nuclear genomes; however, the absence of other WO phage genes in these genomes made this assertion tenuous. Instead, more recent work describing the first WO prophage carrying two sets of CI loci provides compelling evidence for transposon-mediated LGT of CI loci between divergent wolbachiae (Cooper et al., 2019). This phage-independent mechanism for RP gene jumping in wolbachiae may result in the inadvertent capture of these genes by arthropod host genomes.

**Figure 4:**
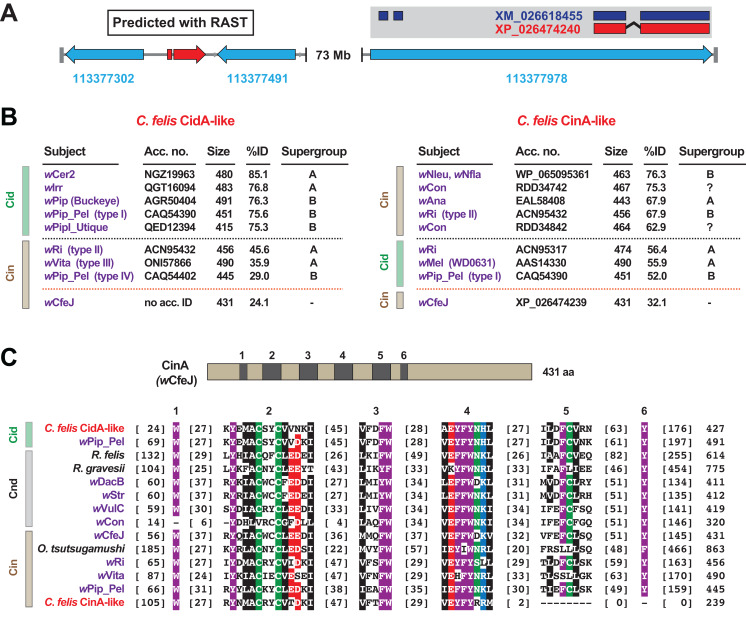
Identification of two CI-like antidote genes in the cat flea genome. (A) The largest *C*. *felis* scaffold (NW_020538040) contains a CidA-like gene, predicted using RAST (Aziz et al., 2008) (left), and a CinA-like exon encoded within a larger gene model with transcriptional support (right). Red, CI-like antidote genes. Blue, *C*. *felis* genes: 113377302 (RING finger and SPRY domain-containing protein 1-like); 113377491 (negative elongation factor B); 113377978 (uncharacterized). The CinA-like exon (XP_026474240) contains a corresponding transcript (dark blue, XM_026618455) generated from a study (Misof et al., 2014) independent of the cat flea genome sequencing (Driscoll et al., 2020). (B) Sequence similarity profiles for the *C*. *felis* CidA-like (left) and CinA-like (right) antidotes. Subjects above the black dashed line are the top hits from a Blastp search against the NCBI nr protein database; subjects below are weaker matches to CinA (left) and CidA (right) proteins; matches to *w*CfeJ CinA are below the orange line. Size, amino acids. (C) Alignment of *C*. *felis* CidA-like and CinA-like antidotes to CidA, CndA and CinA proteins from selected Rickettsiaceae species and *Wolbachia* strains. Schema at top depicts the *w*CfeJ CinA antidote and location of six conserved regions shown in the alignment below. Amino acid coloring as follows: black, hydrophobic; red, negatively charged; green, hydrophilic; purple, aromatic; blue, positively charged. Alignment generated using MUSCLE, default parameters (Edgar, 2004).

When we analyzed the cat flea genome for CI-like genes, we discovered that the largest *C*. *felis* scaffold (185.5 Mb) was found to harbor two CI-like antidote genes (Fig. 4A). Remarkably, neither antidote has any significant similarity to CinA of *w*CfeJ (Fig. 4B), indicating that these CI antidotes originated from other, as yet undescribed wolbachiae. The *C. felis* CinA-like CDS (XP_026474240.1) has greater similarity to CinA proteins than CidA proteins, while the CidA-like CDS (not annotated in the current *C*. *felis* assembly) has much greater similarity to CidA antidotes (Beckmann, Ronau & Hochstrasser, 2017; Beckmann & Fallon, 2013; Bonneau et al., 2018; Madhav et al., 2020; Morrow et al., 2020). The *C*. *felis cinA*-like gene is found as an exon within a larger flea gene model (LOC113377978), and is well supported (18× coverage, 7.2 TPM) by transcripts from the 1KITE project (Misof et al., 2014). Since these transcripts were generated in fleas from a different colony (Dr. Michael Dryden, Kansas State University), we conclude that this gene transfer occurred during an historical infection predating the establishment of these two colonies. Finally, the *C*. *felis* CinA- and CidA-like antidotes each possess the conserved motifs we previously defined for all CI antidotes (Gillespie et al., 2018), with the exception of a truncation of motifs 5 and 6 in the CinA-like protein (Fig. 4C). As other arthropod-encoded antidotes share this C-terminal truncation, it may signify an expendable domain in the host (i.e., a bacterial secretion signal, a motif involved in cognate toxin activation or delivery, etc.).

The incorporation of a *Wolbachia* antidote into an exon of a host gene provides one explanation for how such a laterally transferred gene would remain functional in the absence of the native regulatory elements encoded on WO prophage genomes. We previously hypothesized that CI antidote genes would be under strong selection in arthropod genomes if their expression could result in rescuing CI induction by reproductive parasites (Gillespie et al., 2018). In support of this supposition, it has been shown that: host genotypes modulate CI (Metcalf et al., 2014; Hornett et al., 2006, 2014; Bordenstein, Uy & Werren, 2003); some hosts show weak (*Dmel* and *D*. *suzukii* (Cooper et al., 2017; Conner et al., 2017; Hamm et al., 2014)) or strong (*Culex pipiens* and *D*. *simulans* (Poinsot, Charlat & Merçot, 2003; Merçot & Charlat, 2004)) CI; and a *Wolbachia* strain’s CI can be suppressed when transfected across different hosts (Bordenstein, Uy & Werren, 2003). While none of the arthropod-encoded CI antidotes have been characterized, we propose they impart immunity to toxins secreted by intracellular parasites, curtailing chronic parasite drive into populations. At least four independent evolutionary mechanisms might lead to suppression of CI. Gene family expansion of, and/or overexpression of key factors capable of counteracting CI mechanisms is possible; similarly, it was postulated that evolution in Cid interacting proteins like karyopherin-α or P32 could lead to target site resistance (Beckmann et al., 2019). A third possibility contributing to weakening CI, though not suppression per se, is simply the mutational collapse of the operons themselves due to lack of purifying selection (Martinez et al., 2020; Meany et al., 2019). We supply evidence of a fourth possibility whereby CI might be counteracted by germline expression of acquired CI antidotes. Significantly, our finding raises awareness of a potential barrier to *Wolbachia*-mediated pathogen biocontrol measures.

### Characterization of *w*CfeT and *w*CfeJ infection of cat fleas

**Figure 5:**
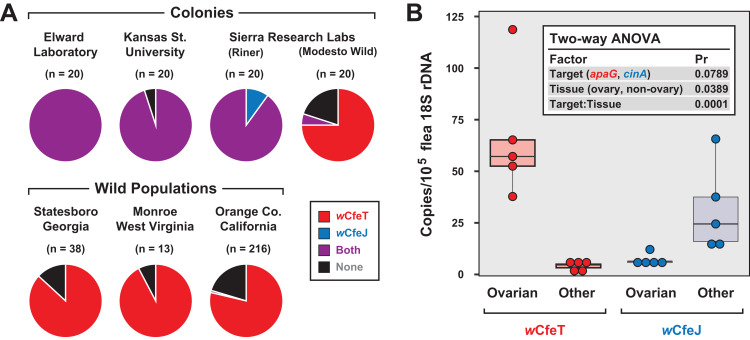
Characterization of *w*CfeJ and *w*CfeJ in cat fleas. (A) Screening for wolbachiae in geographically diverse cat flea colonies (top) and wild populations (bottom). Cat fleas from six locations (including two strains from Sierra Research Laboratories) were assessed for the presence of *w*CfeT and *w*CfeJ. *Wolbachia* infection of individual fleas was assessed by qPCR (see “Materials and Methods” for details). (B) Localization of *w*CfeT and *w*CfeJ in cat flea tissues. Dissected ovaries and other tissues (mostly midguts) from individual cat fleas (EL Colony) were assayed by qPCR (see “Materials and Methods” for details). Five fleas were assayed for each tissue type.

Coupled with earlier reports indicating multiple early-branching wolbachiae lineages infecting *C*. *felis* (Gorham, Fang & Durden, 2003; Casiraghi et al., 2005), our characterization of two divergent *Wolbachia* strains infecting cat fleas in the EL colony (Driscoll et al., 2020) as well as fleas maintained for a decade at LSU (Pornwiroon et al., 2007; Sunyakumthorn et al., 2008; Gillespie et al., 2015) prompted us to test individual fleas for double infection. In addition to EL fleas, three other colony strains and three geographically diverse wild cat flea populations were also surveyed (Fig. 5A). Aside from the Modesto Wild strain, which is historically replenished with wild-caught cat fleas (Dr. B. Donahue, 2017, personal communication), strong *w*CfeJ-*w*CfeT co-infection was found in colony fleas. Conversely, single *w*CfeT infection dominated wild cat fleas, with only a few fleas from a large sampling throughout Orange Co. CA harboring *w*CfeJ. While far greater sampling of both colony and wild populations is necessary, our results hint at a strong selection for *w*CfeT over *w*CfeJ in nature, which is concordant with our premise that *w*CfeT’s BOOM might drive mutualism. The ability of *w*CfeJ to highly infect colony fleas may arise as a consequence of bottlenecks within colonies, which are known to strengthen CI and maternal artificial transmission (Turelli, 1994). No sex ratio distortions were observed in colony or wild fleas, ruling out other forms of RP as driving factors. Nonetheless, the ability of *w*CfeT and *w*CfeJ to co-infect individual cat fleas provides a system to study contrasting forces (nutritional symbiosis and RP) that potentially drive multiple *Wolbachia* infections in a single invertebrate host.

To gain more insight into the nature of the *w*CfeT-*w*CfeJ co-infection, we analyzed the distribution of *w*CfeT and *w*CfeJ in EL flea tissues (Fig. 5B). Surmising that both *Wolbachia* strains are maternally transmitted, we dissected out the ovaries of unfed virgin female fleas and compared *Wolbachia* density in these tissues vs others (primarily midgut). While *w*CfeT predominantly localizes in flea ovaries, the cif carrier, *w*CfeJ, mostly localizes to somatic tissues, although minimal co-localization was observed. The significance of this distribution is unclear. *w*CfeJ may relocate to ovaries upon a mating cue, or perhaps exert CI via some undefined somatic cell mechanism. Transmission of *w*CfeJ may also be predominantly horizontal via environmental or direct flea contact (per (Pietri, DeBruhl & Sullivan, 2016)). Sampling co-infected fleas at specific timepoints during development, as well as determining *w*CfeJ tissue localization in the absence of *w*CfeT infection, will be required to unravel the intertwined transmission dynamics of these *Wolbachia* strains in cat fleas.

### Evolution of *Wolbachia* mutualism and reproductive parasitism

**Figure 6:**
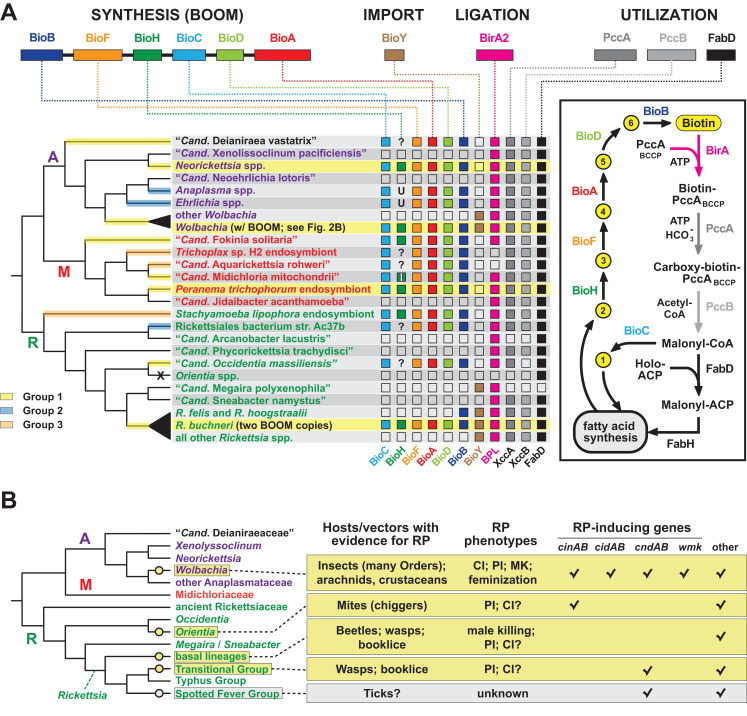
Recurrent evolution of nutritional-mediated mutualism and reproductive parasitism across diverse rickettsial lineages. (A) Diverse strategies for biotin biosynthesis, acquisition and utilization in Rickettsiales. Top, biotin synthesis genes (BOOM arrangement), the BioY biotin transporter, the biotin ligase BirA, and biotin utilization enzymes (biotin-dependent propionyl-CoA carboxylase complex and FabD) involved in malonyl-CoA synthesis. The distribution of these genes across major rickettsial lineages is summarized, with phylogeny of Rickettsiales drawn as a consensus from recent studies (Driscoll et al., 2017; Castelli et al., 2019; Muñoz-Gómez et al., 2019; Szokoli et al., 2016). The acquisition of biotin synthesis operons is depicted by yellow, blue and orange highlighting over branches (see estimated phylogeny in Fig. 1A). Taxa highlighted yellow indicate the conservation of BOOM gene arrangement (see Fig. S3). Question marks indicate missing BioH enzymes in otherwise complete biotin synthesis pathways. These genomes were scanned for other functionally equivalent methyl esterases and found to contain none (Fig. S4D). Inset highlights the gatekeeper role of BioH in shunting X Pimeloyl-CoA methyl ester (metabolite 2) away from fatty acid biosynthesis for the production of biotin. All other metabolites in the biotin synthesis pathway are described in Fig. 2A. X, complete loss of biotin utilization in *O. tsutsugamushi*. (B) Phylogenomic analysis of RP in Rickettsiales. Phylogeny estimation is further simplified relative to the tree in panel A. RP is considered an acquired trait, with origins shown as yellow circles. Spotted Fever Group rickettsiae are shown in gray since RP is unknown, though putative RP-inducing genes are present in some species. Information on rickettsial hosts/vectors (Gillespie et al., 2012) and known RP (Gillespie et al., 2018; Beckmann et al., 2019) are compiled from numerous reports. Check marks reflect the compilations of groups of RP-inducing genes from our prior report, or the recent identification of the *WO-mediated killing* (*wmk*) gene implicated in *Wolbachia* male-killing (Perlmutter et al., 2019). Helix-turn-helix domains of certain XRE-family like proteins across Rickettsiales were not considered related to *wmk*-encoded proteins. “Other” refers to additional proteins presented in our prior report, such as OTU domain-containing proteins and proteins containing the general CndB scaffold (Gillespie et al., 2018).

Order Rickettsiales is a highly diverse group of obligate intracellular bacteria (Gillespie et al., 2012; Driscoll et al., 2013; Darby et al., 2007). A rapid pace of newly identified lineages (Klinges et al., 2019; Castelli et al., 2019; Tashyreva et al., 2018; Kamm et al., 2019; Gruber-Vodicka et al., 2019; Martijn et al., 2015; Schulz et al., 2016; Yurchenko et al., 2018; Schrallhammer et al., 2013; Montagna et al., 2013; Boscaro et al., 2019; Muñoz-Gómez et al., 2019; Mediannikov et al., 2014; Szokoli et al., 2016; Prokopchuk et al., 2019) provides a rich source for evaluating mechanisms of evolution within constraints of the eukaryotic cell. Phage, plasmids, transposons, ICEs, and other insertion sequences are variably present across rickettsial genomes and mobilize numerous genes to offset reductive genome evolution and provide lifestyle altering traits (Gillespie et al., 2012). Our analyses of *Wolbachia* BOOM and RP genes prompted us to evaluate the recurrent evolution of nutritional-mediated mutualism and reproductive parasitism across diverse rickettsial lineages (Fig. 6).

#### Nutritional-mediated mutualism

Phylogeny estimation supports three independent acquisitions of biotin biosynthesis genes by rickettsial species (Fig. 6A; Fig. S1A). The largest clade (group 1) contains all BOOM-containing species, though only *Neorickettsia* spp., certain wolbachiae, *R*. *buchneri*, and the *Peranema trichophorum* endosymbiont carry the conserved BOOM gene arrangement (highlighted yellow in Fig. 6A). Other species in this group, as well as Groups 2 and 3, show different gene arrangements indicating recombination and/or possible replacement of specific *bio* genes through additional LGT (Fig. S3). BOOM and other *bio* gene clusters have repeatedly lost *bioH*, which encodes the methyl esterase generating pimeloyl-CoA, the last step in the synthesis of the pimelate moiety of biotin. Bacteria have evolved numerous strategies for catalyzing this reaction (Lin & Cronan, 2011; Shapiro, Chakravartty & Cronan, 2012; Bi et al., 2016; Feng et al., 2014; Zeng et al., 2020; Chow et al., 2018; Shi et al., 2016; Estrada et al., 2017; Sullivan et al., 2001). Interestingly, a novel methyl esterase of *Ehrlichia chaffeensis* (BioU) was recently shown to complement an *E*. *coli ΔbioH* mutant (Hang et al., 2019); however, we determined that BioU orthologs are present in many rickettsial species, including some that harbor a BioH already and others with no *bio* synthesis genes at all (Figs. S4A–S4C). This indicates that while BioU may generate pimeloyl-CoA in certain rickettsial species—namely, those with complete *bio* synthesis modules except for BioH—it is likely to have a broad range of functions in bacteria (Fig. S4D). Furthermore, some species lack the genes for both BioH and BioU, indicating that alternative pathways for generating pimeloyl-CoA await discovery.

Analysis of the genomic distribution of biotin synthesis genes, the biotin transporter BioY, the biotin ligase BirA, and the enzymes involved in malonyl-CoA synthesis (biotin-dependent propionyl-CoA carboxylase complex and FabD) revealed several strategies for biotin utilization in Rickettsiales (Fig. 6A). Species that harbor a complete set of *bio* genes (as discussed above) also contain ligase and utilization enzymes, although (not surprisingly) most do not carry the BioY importer. Genera *Wolbachia* and *Rickettsia* largely rely on parasitic scavenging of host biotin to fuel fatty acid biosynthesis: they are dominated by species that lack *bio* genes but carry the BioY importer, BirA ligase, and utilization enzymes. Two species of Transitional Group Rickettsiae (*R*. *felis* and *R*. *hoogstraalii*) may be able to utilize imported dethiobiotin as well, as they retain the biotin synthase BioB; alternatively, this may represent the frayed end of a decaying metabolic pathway similar to the glycerol phospholipid and folate biosynthesis pathways in Rickettsiae (Driscoll et al., 2017; Audia, 2012). While characterized as two-component transporters (with BioM/N), BioY subunits alone can efficiently transport biotin into cells (Hebbeln et al., 2007) and also participate with host transporters to relocate cytosolic biotin to vacuoles occupied by pathogens (Fisher et al., 2012). However, certain rickettsial species lacking synthesis genes also lack BioY importers yet retain the ligase and utilization enzymes, indicating the presence of alternative transport mechanisms. Remarkably, *Orientia* spp. lack all genes involved in biotin synthesis, import, ligation and utilization, exhibiting the ultimate form of parasitism for host-dependent fatty acid synthesis: these Rickettsiae must pilfer malonyl-CoA from the host (Nakayama et al., 2008), using a transport system yet to be described.

The acquisition of BOOM by certain wolbachiae and *R*. *buchneri* provides a theoretical framework for how LGT facilitates a transition from metabolite thievery to nutritional-mediated symbiosis. However, it is important to consider that these endoparasites may be utilizing the BOOM primarily for selfish purposes, as almost all bacteria require biotin. For example, *Neorickettsia* spp. and the deltaproteobacterium *Lawsonia intracellularis* are pathogens that also harbor the BOOM, suggesting a more complex ecological role surrounding biotin acquisition. If *Neorickettsia* spp. do indeed actively provision biotin to their trematode and insect vectors, as has been shown for *w*Cle (Nikoh et al., 2014), such host-specific supplementation would necessitate as-yet unidentified genes encoding export factors as well as stringent regulation of the BOOM.

Canonical regulation of biotin synthesis is accomplished via a repressor, fused to the N-terminal domain of the biotin ligase gene, which down-regulates transcription of the *bio* gene cluster in response to high cellular biotin (Barker & Campbell, 1981; Satiaputra et al., 2016). Interestingly, all rickettsial BirA biotin ligases lack this repressor domain, possibly indicating alternative mechanisms for regulating biotin synthesis and host supplementation by these bacteria. “*Candidatus* Aquarickettsia rohweri” and the *Trichoplax* sp. H2 endosymbiont (Midichloriaceae) lack BirA ligases altogether (Fig. 6A), and no other biotin ligases were found in these genomes. Regarding the *Trichoplax* sp. H2 endosymbiont, our prior analysis of the closely related Rickettsiales endosymbiont of *Trichoplax adhaerens* (RETA) discovered two biotin-protein ligase N-terminal domain-containing proteins (BPL_N) encoded in the *T*. *adhaerens* genome (Driscoll et al., 2013). These host-encoded genes contain introns and eukaryotic regulatory elements yet are strongly supported as rickettsial in origin. Homologs of these *T*. *adhaerens* proteins are also present in the *Trichoplax* sp. H2 genome (Kamm et al., 2018) (NCBI acc. nos. RDD42697 and RDD42436), supporting their cross-domain LGT. This raises the possibility for a metabolic interdependence involving biotin synthesis and utilization in placozoans and their rickettsial symbionts, similar to the mosaic metabolic networks underpinning nutritional symbioses in other systems (Bublitz et al., 2019; McCutcheon, 2010; Wilson & Duncan, 2015). For example, the nested tripartite symbiosis between *Planococcus citri* mealybugs, *Tremblaya princeps*, and *Moranella endobia* shows evidence for LGT of several *bio* genes to the mealybug genome (Husnik et al., 2013); these genes share highest similarity to *Wolbachia* BOOM counterparts. This is reminiscent of the pea aphid-*Buchnera* symbiosis, wherein several rickettsial LGTs have been implicated in facilitating metabolic interdependence between host and symbiont (Nikoh et al., 2010). For rickettsial endosymbionts of placozoans, host acquisition of BPL_N genes (among others (Driscoll et al., 2013)) may have established an inextricable obligatory relationship (McCutcheon, 2016; McCutcheon, Boyd & Dale, 2019) and may hint at the evolutionary fate of other BOOM-containing rickettsiae. However, while our analyses of the BOOM and other *bio* genes in rickettsial genomes are suggestive, in the absence of experimental validation we urge caution against implicating these genes as factors underpinning nutritional-mediated mutualism.

#### Reproductive parasitism

Among other bacterial reproductive parasites of arthropods (e.g., species of *Cardinium*, *Arsenophonus* and *Spiroplasma*) wolbachiae are perhaps the best-studied for their roles in RP induction (Hurst & Frost, 2015). Yet within the Rickettsiales, wolbachiae do not walk alone in inducing RP in their hosts, as three other lineages contain species known to induce RP phenotypes (*Orientia tsutsugamushi*, basal *Rickettsia* species and Transitional Group Rickettsiae). Inspection of genomic data indicates SFG Rickettsiae may also harbor reproductive parasites (Fig. 6B). These analyses reveal four discrete molecular determinants for RP induction that have evolved throughout rickettsial evolution (*cinAB*, *cidAB*, *cndAB*, *wmk*), though others likely exist based on previous description of numerous gene models sharing commonalities across toxins and antidote architectures (Gillespie et al., 2018). We posit at least four independent gains of RP in the Rickettsiaceae have occurred, coupled with widespread RP seen in wolbachiae (Fig. 6B). This speculative interpretation is more parsimonious than treating RP as an ancestral rickettsial trait, given that RP-inducing genes strongly associate with mobile genetic elements (e.g., WO prophage in *Wolbachia* strains, RAGE ICEs and plasmids in *Rickettsia* species). Additionally, were RP an ancestral rickettsial trait, one would expect to see cifs in *Rickettsia* genomes more frequently than what is observed based on analysis of available genomes (Gillespie et al., 2018).

In contrast to rickettsiae, RP-inducing genes are found in greater numbers and diversity in wolbachiae, undoubtedly due to their strong association with EAMs of WO prophage genomes and mobile elements (Bordenstein & Bordenstein, 2016; Lindsey et al., 2018). In our previous work, we uncovered a link between the characterized CI-inducing CinA/B and CidA/B operons of wolbachiae and a putative TA operon encoded on the pLbAR plasmid of *Rickettsia felis* str. LSU-Lb, an obligate endosymbiont of the booklouse *Liposcelis bostrychophila* (Gillespie et al., 2015). We posited that this TA operon induces parthenogenesis in the booklouse: pLbAR is unknown in other strains of *R*. *felis* which do not induce parthenogenesis, in booklice or other arthropods (Gillespie et al., 2015), and booklice lacking *R*. *felis* str. LSU-Lb reproduce sexually (Yang et al., 2015; Mockford & Krushelnycky, 2008). Aside from being a CndB toxin (harboring both the NUC and DUB domains of wolbachiae CinB and CidB toxins, respectively) the large pLbAR toxin (3,241 aa) was found to carry several other domains present in other *Wolbachia* proteins, most of which are also EAM-associated. These domains, together with the large central domain (LCD) of the pLbAR toxin, were utilized to identify an extraordinarily diverse array of candidate RP-inducing toxins in a wide-range of intracellular bacteria, some of which are known reproductive parasites (Gillespie et al., 2018). Linked within the intracellular mobilome, we consider this vast family of proteins a source for the recurrent evolution of RP in invertebrate-borne bacteria.

Aside from CI induction by CinA/B and CidA/B operons (Chen et al., 2019; Beckmann et al., 2019; LePage et al., 2017; Beckmann, Ronau & Hochstrasser, 2017), virtually nothing is known about the functions of related candidate RP-inducing genes. The function of the CinA/B-like operon of *O. tsutsugamushi* is unclear. *O. tsutsugamushi* is the pathogen causing scrub typhus and is capable of inducing parthenogenesis in *Leptotrombidium* mites (Roberts, Rapmund & Cadigan, 1977; Takahashi et al., 1997). Given this fact, exploration of its corresponding CinA/B module is warranted. Though the connection to RP in mites is unclear given that CinA/B only occurs in the Boryong strain; a myriad of other proteins encoding the pLbAR LCD are amplified in all *Orientia* genomes. Genome sequences are needed to determine toxin profiles for *Rickettsia* species either in basal lineages (Perotti et al., 2006; Gualtieri et al., 2017; Giorgini et al., 2010) or Transitional Group rickettsiae (Perotti et al., 2006; Hagimori et al., 2006; Nugnes et al., 2015) that induce parthenogenesis in eulophid wasps or trogiid booklice. Genome sequences are also lacking for *Rickettsia* species from basal lineages known to induce male-killing in buprestid and ladybird beetles (Werren et al., 1994; Von der Schulenburg et al., 2001; Lawson et al., 2001; Majerus & Majerus, 2010). We previously mined the genome of the male killer *Rickettsia* parasite of the ladybird beetle *Adalia bipunctata* (Murray et al., 2016) and found a toxin highly similar in architecture to the *Spiroplasma* male killer toxin (Harumoto & Lemaitre, 2018). Both toxins carry an ovarian tumor (OTU) cysteine protease domain (Makarova, Aravind & Koonin, 2000), which is a deubiquitylating enzyme. Further searching with the OTU domain resulted in a hodgepodge of chimeric toxin architectures from bacteria known to induce other RP phenotypes, such as parthenogenesis (*w*Fol (Faddeeva-Vakhrusheva et al., 2017)) and feminization (*w*VulC (Badawi et al., 2018)). Thus, it is clear that additional high-quality (closed) genomes are sorely needed to correlate RP-inducing protein domains and RP phenotypes. New correlations will lead to downstream functional analyses of target’s abilities to induce RP.

A large number of candidate proteins from the pLbAR-derived assemblage occur in species that are not known as reproductive parasites. These include CndB toxins, NUC- and DUB-domain containing proteins that are much larger than characterized CinB and CidB toxins, diverse OTU-domain containing toxins, and especially proteins harboring the LCD that may or may not exhibit toxin-like profiles (Gillespie et al., 2018). Some of these candidate toxins appear to have cognate antidotes, and some genomes carry antidotes only, highlighting the complex diversity of this biological function. Recently, an EAM-encoded transcriptional factor in *w*Mel termed WO-mediated killing (*wmk*) was shown to induce male-specific lethality during early embryogenesis when expressed transgenically in fruit flies (Perlmutter et al., 2019). This male-killer gene was not detected as part of our pLbAR-derived pool, indicating that RP-inducing gene arsenals are probably much larger than have been described thus far, particularly considering that *cin*, *cid* and *cnd* loci are not present in the genomes of *Cardinium* and *Arsenophonus* reproductive parasites (Gillespie et al., 2018; Beckmann et al., 2019; Gebiola et al., 2017) for which the mechanisms for RP induction remain unknown (Siozios et al., 2019; Duron et al., 2008).

Candidate RP-inducing toxins with similar features to the pLbAR toxin (large, modular, and associated with mobile genetic elements) are found in several *Wolbachia* genomes (e.g., *w*Fol, *w*Str, *w*DacB, *w*VulC, *w*Con, *w*Ced, and *w*CauA). They are often in conjunction with other *cin* and/or *cid* loci, yet not always associated with WO prophage. Consistent with prior reports identifying LGT between *Rickettsia* and *Wolbachia* genomes (Gillespie et al., 2012, 2015; Ishmael et al., 2009; Baldridge et al., 2016), similar toxins in *Rickettsia* genomes (*R*. *felis* str. LSU-Lb, *R*. *gravesii*, male killer of *Adalia bipunctata*) indicate a dynamic platform for disseminating RP-inducing genes across diverse rickettsial species that includes *Rickettsia* plasmids and WO prophage. Growing numbers of identified *Rickettsia* plasmids (Gillespie et al., 2015), proliferative RAGE ICEs in *Rickettsia* and *Orientia* genomes (Gillespie et al., 2012; Hagen et al., 2018; Nakayama et al., 2008; Batty et al., 2018; Cho et al., 2007; Nakayama et al., 2010), and the recent discovery of the first *Wolbachia* plasmid (Reveillaud et al., 2019), attest to a highly dynamic mobilome not previously realized for obligate intracellular bacteria. The proposed fission of a CndB-like toxin into a *w*CfeJ CinB toxin, described in the current study (Fig. 3D), suggests larger toxins undergo streamlining into discrete inducers of CI or other RP phenotypes. As with the evolution of biotin utilization, Rickettsiales provides a framework to study the nature of RP in a diverse array of bacteria that enjoy different relationships with their invertebrate and vertebrate hosts.

## Conclusion

The remarkable opposing features of *w*CfeT and *w*CfeJ offer testable hypotheses for contrasting host relationships (nutritional symbiosis vs RP, respectively). They also provide a framework to study the dynamics of *Wolbachia* co-infections, both in colonies and wild populations. The *w*CfeT and *w*CfeJ genomes yield lessons on the role of LGT in shaping host associations, highlighting the ephemeral nature of nutritional-based mutualism and RP over a general rickettsial platform of obligate intracellular parasitism. Host genome sequences provide clues to on-going mutualism and historical RP, and our hypothesis for host CI-gene capture leading to suppression of RP illuminates possible limitations for deployment of *Wolbachia* reproductive parasites to combat arthropod transmission of human pathogens. In summary, our robust characterization of two divergent strains of *C*. *felis*-associated wolbachiae, as well as the *C. felis* genome itself (Driscoll et al., 2020), provides essential information for determining the impact of these bacteria on cat flea biology and pathogen vectoral dynamics.

## Supplemental Information

10.7717/peerj.10646/supp-1 Supplemental Information 1 Phylogeny estimations of biotin synthesis enzymes and EamA transporters. (**A**) Complete phylogeny estimation of BOOM and other *bio* gene sets from diverse bacteria. Tree was estimated from the concatenation of six *bio* enzymes (BioC, BioH, BioF, BioA, BioD, and BioB) or subsets in certain cases (see **Table S2** for all sequence information and **Materials and Methods** for details on dataset processing and tree estimation). Branch support was assessed with 1,000 pseudo-replications. Final ML optimization likelihood was -127140.955066. In taxon color scheme, [R] denotes Rickettsiales, with Holosporaceae [H] as a revised family of Rhodospirillales (101). The families of Rickettsiales are similarly noted: Anaplasmataceae [A], Midichloriaceae [M], Rickettsiaceae [R], and “*Candidatus* Deianiaeaceae” [D], with the latter considered provisional (91). (**B**) Estimated phylogeny of EamA transporters. See **Materials and Methods** for details on dataset processing and tree estimation. Branch support was assessed with 1,000 pseudo-replications. Final ML optimization likelihood was -23058.797227. Taxon color scheme as described in panel **A**.

10.7717/peerj.10646/supp-2 Supplemental Information 2 In silico characterization of the *w* CfeT prophage genome. Schema shows the comparison of the *w*CfeT and WOVitA1 prophage genomes, with gene descriptions following prior studies (26, 30, 148, 183). *w*CfeT genes within the typical Eukaryotic Association Module (EAM) were used as queries in Blastp searches against the NCBI nr protein database, with summary statistics provided for top subjects (dashed box).

10.7717/peerj.10646/supp-3 Supplemental Information 3 Synteny analysis of biotin synthesis genes in Rickettsiales genomes. Rickettsiales taxa with at least 5 of 6 biotin synthesis genes (n=28) were analyzed for co-linearity of their biotin genes. Ortholog groups (n=2,527) were constructed from these genomes as described for **Fig. 1** (see **Materials and Methods**) and OGs containing biotin synthesis genes identified. Genome locations were retrieved from NCBI in gene file format (gff) and used to order the orthologs in each genome. For unclosed genomes, all contigs were included in OG construction; only a single genome (*Candidatus* Aquarickettsia rohweri) contained biotin synthesis genes on multiple contigs. Biotin genes are colored according to the ideal BOOM shown at the top. Black boxes indicate stretches of unrelated genes. Taxa are grouped into 3 classes according to the synteny of biotin genes in their genomes: Class I taxa demonstrate completely conserved gene order; Class II taxa contain only a single gene out of order (Class IIA: BioA; Class IIB: BioB); Class III taxa exhibit little or no conservation of gene order. All blocks are anchored on BioB for ease of comparison, except Class IIB which are anchored on BioF instead.

10.7717/peerj.10646/supp-4 Supplemental Information 4 Bioinformatics analysis of BioU and related methyl esterases. (**A**) Alignment of *Ehrlichia chaffeensis* and *Anaplasma centrale* BioU proteins with orthologs from other Rickettsiales species. BioU orthologs were retrieved from Blastp searches against the NCBI Rickettsiales databse using *E*. *chaffeensis* str. Sapulpa BioU (EAM85518.1) as a query. The catalytic residues (Ser–Asp–His) characteristic of methyl esterases are highlighted yellow (184). Sequences were aligned with MUSCLE v3.8.31 (169) using default parameters. NCBI protein accession numbers are listed in panel **C**. (**B**) Percent identity matrix calculated from the alignment shown in panel **A**. (**C**) Estimated phylogeny of proteins shown in panel **A**. Two alphaproteobacterial outgroups and two hymenopteran mitochondrial methyl esterases are included. Phylogeny was estimated using RAxML v8.2.4 (171) under the gamma model of rate heterogeneity and estimation of the proportion of invariant sites. Branch support was assessed with 1,000 pseudo-replications. Final ML optimization likelihood was -5448.767799. (**D**) Analysis of eight rickettsial genomes that contain biotin synthesis genes except for the BioH methyl esterase. Blastp searches against the rickettsial genomes were conducted using the following queries, all of which have been shown to orthogonally substitute for BioH: BioV of *Helicobacter* sp. L8b (TSA87024.1); BioG of *Haemophilus influenzae* str. CGSHiCZ412602 (AIB45959.1); BtsA of *Moraxella catarrhalis* str. CCUG 353 (KZR94829.1); BioJ of *Francisella marina* str. E103-15 (QEO56965.1); BioZ of *Mesorhizobium japonicum* str. R7A (AAG47795.1); EstN1 of *Nitrososphaera viennensis* str. EN76 (AIC14436.1); BioK of *Prochlorococcus marinus* str. SS51 (KGG31655.1).

10.7717/peerj.10646/supp-5 Supplemental Information 5 Information supporting the *Wolbachia* genome-based phylogeny estimation.

10.7717/peerj.10646/supp-6 Supplemental Information 6 Information supporting the phylogeny estimation of biotin genes.

10.7717/peerj.10646/supp-7 Supplemental Information 7 Information supporting the comparative analysis of BOOM gene neighborhoods in wolbachiae genomes.

## Additional Information and Declarations

### DNA Deposition

The following information was supplied regarding the deposition of DNA sequences: All relevant information pertaining to wCfeT and wCfeJ is available at NCBI: PRJNA622233. Complete genome sequences are available in the NCBI RefSeq database: NZ_CP051156.1 (wCfeT) and NZ_CP051157.1 (wCfeJ).

### Data Availability

The following information was supplied regarding data availability: Data generated for this project, including raw PCR data for Fig. 5, are available at GitHub: https://www.github.com/wvuvectors/wCfe_genomes.

## References

[ref-1] Aikawa T, Anbutsu H, Nikoh N, Kikuchi T, Shibata F, Fukatsu T (2009). Longicorn beetle that vectors pinewood nematode carries many Wolbachia genes on an autosome. Proceedings of the Royal Society B: Biological Sciences.

[ref-2] Angelakis E, Mediannikov O, Parola P, Raoult D (2016). Rickettsia felis: the complex journey of an emergent human pathogen. Trends in Parasitology.

[ref-3] Audia JP, Palmer GH, Azad AF (2012). Physiology and metabolism in the face of reductive evolution. Intracellular Pathogens II: Rickettsiales.

[ref-4] Augustinos AA, Santos-Garcia D, Dionyssopoulou E, Moreira M, Papapanagiotou A, Scarvelakis M, Doudoumis V, Ramos S, Aguiar AF, Borges PAV, Khadem M, Latorre A, Tsiamis G, Bourtzis K, Cordaux R (2011). Detection and characterization of Wolbachia infections in natural populations of aphids: is the hidden diversity fully unraveled?. PLOS ONE.

[ref-186] Aziz RK, Bartels D, Best AA, DeJongh M, Disz T, Edwards RA, Formsma K, Gerdes S, Glass EM, Kubal M, Meyer F, Olsen GJ, Olson R, Osterman AL, Overbeek RA, McNeil LK, Paarmann D, Paczian T, Parrello B, Pusch GD, Reich C, Stevens R, Vassieva O, Vonstein V, Wilke A, Zagnitko O (2008). The RAST server: rapid annotations using subsystems technology. BMC Genomics.

[ref-5] Badawi M, Moumen B, Giraud I, Grève P, Cordaux R (2018). Investigating the molecular genetic basis of cytoplasmic sex determination caused by wolbachia endosymbionts in terrestrial isopods. Genes.

[ref-6] Baldo L, Werren JH (2007). Revisiting Wolbachia supergroup typing based on WSP: spurious lineages and discordance with MLST. Current Microbiology.

[ref-7] Baldridge GD, Markowski TW, Witthuhn BA, Higgins L, Baldridge AS, Fallon AM (2016). The Wolbachia WO bacteriophage proteome in the Aedes albopictus C/wStr1 cell line: evidence for lytic activity? Vitr Cell Dev Biol. In Vitro Cellular and Developmental Biology—Animal.

[ref-8] Balvín O, Roth S, Talbot B, Reinhardt K (2018). Co-speciation in bedbug Wolbachia parallel the pattern in nematode hosts. Scientific Reports.

[ref-9] Bandi C, Anderson TJC, Genchi C, Blaxter ML (1998). Phylogeny of Wolbachia in filarial nematodes. Proceedings of the Royal Society of London. Series B: Biological Sciences.

[ref-10] Barker DF, Campbell AM (1981). Genetic and biochemical characterization of the birA gene and its product: evidence for a direct role of biotin holoenzyme synthetase in repression of the biotin operon in Escherichia coli. Journal of Molecular Biology.

[ref-11] Batty EM, Chaemchuen S, Blacksell S, Richards AL, Paris D, Bowden R, Chan C, Lachumanan R, Day N, Donnelly P, Chen S, Salje J (2018). Long-read whole genome sequencing and comparative analysis of six strains of the human pathogen Orientia tsutsugamushi. PLOS Neglected Tropical Diseases.

[ref-12] Beard CB, Butler JF, Hall DW (1990). Prevalence and biology of endosymbionts of fleas (Siphonaptera: Pulicidae) from dogs and cats in Alachua County, Florida. Journal of Medical Entomology.

[ref-13] Beckmann JF, Bonneau M, Chen H, Hochstrasser M, Poinsot D, Merçot H, Weill M, Sicard M, Charlat S (2019). The toxin-antidote model of cytoplasmic incompatibility: genetics and evolutionary implications. Trends in Genetics.

[ref-14] Beckmann JF, Fallon AM (2013). Detection of the Wolbachia protein WPIP0282 in mosquito spermathecae: implications for cytoplasmic incompatibility. Insect Biochemistry and Molecular Biology.

[ref-15] Beckmann JF, Ronau JA, Hochstrasser M (2017). A Wolbachia deubiquitylating enzyme induces cytoplasmic incompatibility. Nature Microbiology.

[ref-16] Beckmann JF, Sharma GD, Mendez L, Chen H, Hochstrasser M (2019). The Wolbachia cytoplasmic incompatibility enzyme CIDB targets nuclear import and protamine-histone exchange factors. Elife.

[ref-17] Bi H, Zhu L, Jia J, Cronan JE (2016). A biotin biosynthesis gene restricted to helicobacter. Scientific Reports.

[ref-18] Bing XL, Xia WQ, Gui JD, Yan GH, Wang XW, Liu SS (2014). Diversity and evolution of the Wolbachia endosymbionts of Bemisia (Hemiptera: Aleyrodidae) whiteflies. Ecology and Evolution.

[ref-19] Bonneau M, Landmann F, Labbé P, Justy F, Weill M, Sicard M (2018). The cellular phenotype of cytoplasmic incompatibility in Culex pipiens in the light of cidB diversity. PLOS Pathogens.

[ref-20] Bordenstein SR, Bordenstein SR (2016). Eukaryotic association module in phage WO genomes from Wolbachia. Nature Communications.

[ref-21] Bordenstein SR, Uy JJ, Werren JH (2003). Host genotype determines cytoplasmic incompatibility type in the haplodiploid genus Nasonia. Genetics.

[ref-22] Boscaro V, Husnik F, Vannini C, Keeling PJ (2019). Symbionts of the ciliate Euplotes : diversity, patterns and potential as models for bacteria-eukaryote endosymbioses. Proceedings of the Royal Society B: Biological Sciences.

[ref-23] Brianti E, Gaglio G, Napoli E, Giannetto S, Dantas-Torres F, Bain O, Otranto D (2012). New insights into the ecology and biology of Acanthocheilonema reconditum (Grassi, 1889) causing canine subcutaneous filariosis. Parasitology.

[ref-24] Bublitz DAC, Chadwick GL, Magyar JS, Sandoz KM, Brooks DM, Mesnage S, Ladinsky MS, Garber AI, Bjorkman PJ, Orphan VJ, McCutcheon JP (2019). Peptidoglycan production by an insect-bacterial mosaic. Cell.

[ref-25] Capella-Gutiérrez S, Silla-Martínez JM, Gabaldón T (2009). trimAl: a tool for automated alignment trimming in large-scale phylogenetic analyses. Bioinformatics.

[ref-26] Casiraghi M, Bordenstein SR, Baldo L, Lo N, Beninati T, Wernegreen JJ, Werren JH, Bandi C (2005). Phylogeny of Wolbachia pipientis based on gltA, groEL and ftsZ gene sequences: clustering of arthropod and nematode symbionts in the F supergroup, and evidence for further diversity in the Wolbachia tree. Microbiology.

[ref-27] Castelli M, Sabaneyeva E, Lanzoni O, Lebedeva N, Floriano AM, Gaiarsa S, Benken K, Modeo L, Bandi C, Potekhin A, Sassera D, Petroni G (2019). Deianiraea, an extracellular bacterium associated with the ciliate Paramecium, suggests an alternative scenario for the evolution of Rickettsiales. ISME Journal.

[ref-28] Chafee ME, Funk DJ, Harrison RG, Bordenstein SR (2010). Lateral phage transfer in obligate intracellular bacteria (Wolbachia): verification from natural populations. Molecular Biology and Evolution.

[ref-29] Chen H, Ronau JA, Beckmann JF, Hochstrasser M (2019). A Wolbachia nuclease and its binding partner comprise a novel mechanism for induction of cytoplasmic incompatibility. Proceedings of the National Academy of Sciences.

[ref-30] Cho N-H, Kim H-R, Lee J-H, Kim S-Y, Kim J, Cha S, Kim S-Y, Darby AC, Fuxelius H-H, Yin J, Kim JH, Kim J, Lee SJ, Koh Y-S, Jang W-J, Park K-H, Andersson SGE, Choi M-S, Kim I-S (2007). The Orientia tsutsugamushi genome reveals massive proliferation of conjugative type IV secretion system and host cell interaction genes. Proceedings of the National Academy of Sciences.

[ref-31] Chow J, Danso D, Ferrer M, Streit WR (2018). The Thaumarchaeon N. gargensis carries functional bioABD genes and has a promiscuous E. coli ΔbioH-complementing esterase EstN1. Scientific Reports.

[ref-32] Chung M, Munro JB, Tettelin H, Hotopp JCD (2018). Using core genome alignments to assign bacterial species. MSystems.

[ref-33] Conner WR, Blaxter ML, Anfora G, Ometto L, Rota-Stabelli O, Turelli M (2017). Genome comparisons indicate recent transfer of wRi-like Wolbachia between sister species Drosophila suzukii and D. subpulchrella. Ecology and Evolution.

[ref-34] Cooper BS, Ginsberg PS, Turelli M, Matute DR (2017). Wolbachia in the *Drosophila yakuba* complex: pervasive frequency variation and weak cytoplasmic incompatibility, but no apparent effect on reproductive isolation. Genetics.

[ref-35] Cooper BS, Vanderpool D, Conner WR, Matute DR, Turelli M (2019). *Wolbachia* acquisition by *Drosophila yakuba*—clade hosts and transfer of incompatibility loci between distantly related Wolbachia. Genetics.

[ref-36] Darby AC, Cho N-H, Fuxelius H-H, Westberg J, Andersson SGE (2007). Intracellular pathogens go extreme: genome evolution in the Rickettsiales. Trends in Genetics.

[ref-37] Dhaygude K, Nair A, Johansson H, Wurm Y, Sundström L (2019). The first draft genomes of the ant Formica exsecta, and its Wolbachia endosymbiont reveal extensive gene transfer from endosymbiont to host. BMC Genomics.

[ref-38] Dittmar K, Whiting MF (2004). New Wolbachia endosymbionts from Nearctic and Neotropical fleas (Siphonaptera). Journal of Parasitology.

[ref-39] Douglas AE (2015). Multiorganismal insects: diversity and function of resident microorganisms. Annual Review of Entomology.

[ref-40] Driscoll TP, Gillespie JJ, Nordberg EK, Azad AF, Sobral BW (2013). Bacterial DNA sifted from the Trichoplax adhaerens (Animalia: Placozoa) genome project reveals a putative rickettsial endosymbiont. Genome Biology and Evolution.

[ref-41] Driscoll TP, Verhoeve VI, Gillespie J, Johnston JS, Guillotte ML, Rennoll-Bankert KE, Rahman MS, Hagen D, Elsik CG, Macaluso K, Azad A (2020). Cat fleas in flux: rampant gene duplication, genome size plasticity, and paradoxical Wolbachia infection. BMC Biology.

[ref-42] Driscoll TP, Verhoeve VI, Guillotte ML, Lehman SS, Rennoll SA, Beier-Sexton M, Rahman MS, Azad AF, Gillespie JJ (2017). Wholly *Rickettsia*! Reconstructed metabolic profile of the quintessential bacterial parasite of eukaryotic cells. MBio.

[ref-43] Duron O, Bouchon D, Boutin S, Bellamy L, Zhou L, Engelstädter J, Hurst GD (2008). The diversity of reproductive parasites among arthropods: wolbachia do not walk alone. BMC Biology.

[ref-44] Edgar RC (2004). MUSCLE: multiple sequence alignment with high accuracy and high throughput. Nucleic Acids Research.

[ref-45] Estrada P, Manandhar M, Dong SH, Deveryshetty J, Agarwal V, Cronan JE, Nair SK (2017). The pimeloyl-CoA synthetase BioW defines a new fold for adenylate-forming enzymes. Nature Chemical Biology.

[ref-46] Faddeeva-Vakhrusheva A, Kraaijeveld K, Derks MFL, Anvar SY, Agamennone V, Suring W, Kampfraath AA, Ellers J, Le Ngoc G, van Gestel CAM, Mariën J, Smit S, Van Straalen NM, Roelofs D (2017). Coping with living in the soil: the genome of the parthenogenetic springtail Folsomia candida. BMC Genomics.

[ref-47] Feng Y, Napier BA, Manandhar M, Henke SK, Weiss DS, Cronan JE (2014). A Francisella virulence factor catalyses an essential reaction of biotin synthesis. Molecular Microbiology.

[ref-48] Fisher DJ, Fernández RE, Adams NE, Maurelli AT (2012). Uptake of biotin by chlamydia spp. through the use of a bacterial transporter (BioY) and a host-cell transporter (SMVT). PLOS ONE.

[ref-49] Fourie JJ, Crafford D, Horak IG, Stanneck D (2012). Prophylactic treatment of flea-infested cats with an imidacloprid/flumethrin collar to forestall infection with Dipylidium caninum. Parasites & Vectors.

[ref-50] Gebiola M, Giorgini M, Kelly SE, Doremus MR, Ferree PM, Hunter MS (2017). Cytological analysis of cytoplasmic incompatibility induced by Cardinium suggests convergent evolution with its distant cousin Wolbachia. Proceedings of the Royal Society B: Biological Sciences.

[ref-51] Gerth M, Bleidorn C (2017). Comparative genomics provides a timeframe for Wolbachia evolution and exposes a recent biotin synthesis operon transfer. Nature Microbiology.

[ref-52] Gillespie JJ, Driscoll TP, Verhoeve VI, Rahman MS, Macaluso KR, Azad AF (2018). A tangled web: origins of reproductive parasitism. Genome Biology and Evolution.

[ref-53] Gillespie JJ, Driscoll TP, Verhoeve VI, Utsuki T, Husseneder C, Chouljenko VN, Azad AF, Macaluso KR (2015). Genomic diversification in strains of rickettsia felis isolated from different arthropods. Genome Biology and Evolution.

[ref-54] Gillespie JJ, Joardar V, Williams KP, Driscoll TP, Hostetler JB, Nordberg E, Shukla M, Walenz B, Hill CA, Nene VM, Azad AF, Sobral BW, Caler E (2012). A Rickettsia genome overrun by mobile genetic elements provides insight into the acquisition of genes characteristic of an obligate intracellular lifestyle. Journal of Bacteriology.

[ref-55] Gillespie JJ, Nordberg EK, Azad AA, Sobral BW, Azad AF, Palmer GH (2012). Phylogeny and comparative genomics: the shifting landscape in the genomics era. Intracellular Pathogens II: Rickettsiales.

[ref-56] Giorgini M, Bernardo U, Monti MM, Nappo AG, Gebiola M (2010). Rickettsia symbionts cause parthenogenetic reproduction in the parasitoid wasp Pnigalio soemius (Hymenoptera: Eulophidae). Applied and Environmental Microbiology.

[ref-57] Glickman LT, Moore GE, Glickman NW, Caldanaro RJ, Aucoin D, Lewis HB (2006). Purdue university-banfield national companion animal surveillance program for emerging and zoonotic diseases. Vector-Borne Zoonotic Diseases.

[ref-58] Glowska E, Dragun-Damian A, Dabert M, Gerth M (2015). New Wolbachia supergroups detected in quill mites (Acari: Syringophilidae). Infection Genetics and Evolution.

[ref-59] González-Álvarez VH, De Mera IGF, Cabezas-Cruz A, De la Fuente J, Ortega-Morales AI, Almazán C (2017). Molecular survey of Rickettsial organisms in ectoparasites from a dog shelter in Northern Mexico. Veterinary Parasitology: Regional Studies and Reports.

[ref-60] Gorham CH, Fang QQ, Durden LA (2003). Wolbachia endosymbionts in fleas (Siphonaptera). Journal of Parasitology.

[ref-61] Gruber-Vodicka HR, Leisch N, Kleiner M, Hinzke T, Liebeke M, McFall-Ngai M, Hadfield MG, Dubilier N (2019). Two intracellular and cell type-specific bacterial symbionts in the placozoan Trichoplax H2. Nature Microbiology.

[ref-62] Gualtieri L, Nugnes F, Nappo AG, Gebiola M, Bernardo U (2017). Life inside a gall: closeness does not favour horizontal transmission of Rickettsia between a gall wasp and its parasitoid. FEMS Microbiology Ecology.

[ref-63] Gómez-Valero L, Soriano-Navarro M, Pérez-Brocal V, Heddi A, Moya A, García-Verdugo JM, Latorre A (2004). Coexistence of Wolbachia with Buchnera aphidicola and a secondary symbiont in the aphid Cinara cedri. Journal of Bacteriology.

[ref-64] Haegeman A, Vanholme B, Jacob J, Vandekerckhove TTM, Claeys M, Borgonie G, Gheysen G (2009). An endosymbiotic bacterium in a plant-parasitic nematode: member of a new Wolbachia supergroup. International Journal for Parasitology.

[ref-65] Haferkamp I, Penz T, Geier M, Ast M, Mushak T, Horn M, Schmitz-Esser S (2013). The endosymbiont amoebophilus asiaticus encodes an s-adenosylmethionine carrier that compensates for its missing methylation cycle. Journal of Bacteriology.

[ref-66] Hagen R, Verhoeve VI, Gillespie JJ, Driscoll TP (2018). Conjugative transposons and their cargo genes vary across natural populations of Rickettsia buchneri infecting the tick Ixodes scapularis. Genome Biology and Evolution.

[ref-67] Hagimori T, Abe Y, Date S, Miura K (2006). The first finding of a Rickettsia bacterium associated with parthenogenesis induction among insects. Current Microbiology.

[ref-68] Hamm CA, Begun DJ, Vo A, Smith CCR, Saelao P, Shaver AO, Jaenike J, Turelli M (2014). *Wolbachia* do not live by reproductive manipulation alone: infection polymorphism in *Drosophila suzukii* and D. subpulchrella. Molecular Ecology.

[ref-69] Hang X, Zeng Q, Zeng L, Jia J, Bi H (2019). Functional replacement of the bioc and bioh proteins of escherichia coli biotin precursor biosynthesis by ehrlichia chaffeensis novel proteins. Current Microbiology.

[ref-70] Harumoto T, Lemaitre B (2018). Male-killing toxin in a bacterial symbiont of Drosophila. Nature.

[ref-71] Hebbeln P, Rodionov DA, Alfandega A, Eitinger T (2007). Biotin uptake in prokaryotes by solute transporters with an optional ATP-binding cassette-containing module. Proceedings of the National Academy of Sciences.

[ref-72] Hellemans S, Kaczmarek N, Marynowska M, Calusinska M, Roisin Y, Fournier D (2019). Bacteriome-associated Wolbachia of the parthenogenetic termite Cavitermes tuberosus. FEMS Microbiology Ecology.

[ref-73] Hilgenboecker K, Hammerstein P, Schlattmann P, Telschow A, Werren JH (2008). How many species are infected with Wolbachia? A statistical analysis of current data. FEMS Microbiology Letters.

[ref-74] Hornett EA, Charlat S, Duplouy AMR, Davies N, Roderick GK, Wedell N, Hurst GDD (2006). Evolution of male-killer suppression in a natural population. PLOS Biology.

[ref-75] Hornett EA, Moran B, Reynolds LA, Charlat S, Tazzyman S, Wedell N, Jiggins CD, Hurst GDD (2014). The evolution of sex ratio distorter suppression affects a 25 cm genomic region in the butterfly hypolimnas bolina. PLOS Genetics.

[ref-76] Hosokawa T, Koga R, Kikuchi Y, Meng XY, Fukatsu T (2010). Wolbachia as a bacteriocyte-associated nutritional mutualist. Proceedings of the National Academy of Sciences.

[ref-77] Hotopp JCD, Clark ME, Oliveira DCSG, Foster JM, Fischer P, Torres MCM, Giebel JD, Kumar N, Ishmael N, Wang S, Ingram J, Nene RV, Shepard J, Tomkins J, Richards S, Spiro DJ, Ghedin E, Slatko BE, Tettelin H, Werren JH (2007). Widespread lateral gene transfer from intracellular bacteria to multicellular eukaryotes. Science.

[ref-78] Hurst GDD, Frost CL (2015). Reproductive parasitism: maternally inherited symbionts in a biparental world. Cold Spring Harbor Perspectives in Biology.

[ref-79] Husnik F, Nikoh N, Koga R, Ross L, Duncan RP, Fujie M, Tanaka M, Satoh N, Bachtrog D, Wilson ACC, Von Dohlen CD, Fukatsu T, McCutcheon JP (2013). Horizontal gene transfer from diverse bacteria to an insect genome enables a tripartite nested mealybug symbiosis. Cell.

[ref-80] Ishmael N, Hotopp JCD, Loanidis P, Biber S, Sakamoto J, Siozios S, Nene V, Werren J, Boutriz K, Bordenstein SR, Tettelin H (2009). Extensive genomic diversity of closely related wolbachia strains. Microbiology.

[ref-81] Jiménez NE, Gerdtzen ZP, Olivera-Nappa Á, Salgado JC, Conca C (2019). A systems biology approach for studying Wolbachia metabolism reveals points of interaction with its host in the context of arboviral infection. PLOS Neglected Tropical Diseases.

[ref-82] Ju J-F, Bing X-L, Zhao D-S, Guo Y, Xi Z, Hoffmann AA, Zhang K-J, Huang H-J, Gong J-T, Zhang X, Hong X-Y (2019). Wolbachia supplement biotin and riboflavin to enhance reproduction in planthoppers. ISME Journal.

[ref-83] Kamm K, Osigus HJ, Stadler PF, DeSalle R, Schierwater B (2018). Trichoplax genomes reveal profound admixture and suggest stable wild populations without bisexual reproduction. Scientific Reports.

[ref-84] Kamm K, Osigus HJ, Stadler PF, DeSalle R, Schierwater B (2019). Genome analyses of a placozoan rickettsial endosymbiont show a combination of mutualistic and parasitic traits. Scientific Reports.

[ref-85] Kampfraath AA, Klasson L, Anvar SY, Vossen RHAM, Roelofs D, Kraaijeveld K, Ellers J (2019). Genome expansion of an obligate parthenogenesis-associated Wolbachia poses an exception to the symbiont reduction model. BMC Genomics.

[ref-86] Kelley LA, Sternberg MJE (2009). Protein structure prediction on the web: a case study using the Phyre server. Nature Protocols.

[ref-87] Kinch LN, Ginalski K, Rychlewski L, Grishin NV (2005). Identification of novel restriction endonuclease-like fold families among hypothetical proteins. Nucleic Acids Research.

[ref-88] Klinges JG, Rosales SM, McMinds R, Shaver EC, Shantz AA, Peters EC, Eitel M, Wörheide G, Sharp KH, Burkepile DE, Silliman BR, Vega Thurber RL (2019). Phylogenetic, genomic, and biogeographic characterization of a novel and ubiquitous marine invertebrate-associated Rickettsiales parasite, Candidatus Aquarickettsia rohweri, gen. nov., sp. nov. ISME Journal.

[ref-89] Kondo N, Nikoh N, Ijichi N, Shimada M, Fukatsu T (2002). Genome fragment of Wolbachia endosymbiont transferred to X chromosome of host insect. Proceedings of the National Academy of Sciences.

[ref-90] Lawrence AL, Hii S-F, Chong R, Webb CE, Traub R, Brown G, Šlapeta J (2015). Evaluation of the bacterial microbiome of two flea species using different DNA-isolation techniques provides insights into flea host ecology. FEMS Microbiology Ecology.

[ref-91] Lawson ET, Mousseau TA, Klaper R, Hunter MD, Werren JH (2001). Rickettsia associated with male-killing in a buprestid beetle. Heredity.

[ref-92] Leclercq S, Thézé J, Chebbi MA, Giraud I, Moumen B, Ernenwein L, Grève P, Gilbert C, Cordaux R (2016). Birth of a W sex chromosome by horizontal transfer of Wolbachia bacterial symbiont genome. Proceedings of the National Academy of Sciences.

[ref-93] Lefoulon E, Bain O, Makepeace BL, D’Haese C, Uni S, Martin C, Gavotte L (2016). Breakdown of coevolution between symbiotic bacteria Wolbachia and their filarial hosts. PeerJ.

[ref-94] Lefoulon E, Clark T, Borveto F, Perriat-Sanguinet M, Moulia C, Slatko BE, Gavotte L (2020). Pseudoscorpion Wolbachia symbionts: diversity and evidence for a new supergroup S. BMC Microbiology.

[ref-95] Lefoulon E, Clark T, Guerrero R, Cañizales I, Cardenas-Callirgos JM, Junker K, Vallarino-Lhermitte N, Makepeace BL, Darby AC, Foster JM, Martin C, Slatko BE (2020). Diminutive, degraded but dissimilar: wolbachia genomes from filarial nematodes do not conform to a single paradigm. BioRxiv.

[ref-97] LePage DP, Metcalf JA, Bordenstein SR, On J, Perlmutter JI, Shropshire JD, Layton EM, Funkhouser-Jones LJ, Beckmann JF, Bordenstein SR (2017). Prophage WO genes recapitulate and enhance Wolbachia-induced cytoplasmic incompatibility. Nature.

[ref-98] Letunic I, Bork P (2017). 20 years of the SMART protein domain annotation resource. Nucleic Acids Research.

[ref-99] Leulmi H, Socolovschi C, Laudisoit A, Houemenou G, Davoust B, Bitam I, Raoult D, Parola P (2014). Detection of *Rickettsia felis*, *Rickettsia typhi*, *Bartonella* Species and *Yersinia pestis* in Fleas (Siphonaptera) from Africa. PLOS Neglected Tropical Diseases.

[ref-100] Li L, Stoeckert CJ, Roos DS (2003). OrthoMCL: identification of ortholog groups for eukaryotic genomes. Genome Research.

[ref-101] Lin S, Cronan JE (2011). Closing in on complete pathways of biotin biosynthesis. Molecular BioSystems.

[ref-102] Lin S, Hanson RE, Cronan JE (2010). Biotin synthesis begins by hijacking the fatty acid synthetic pathway. Nature Chemical Biology.

[ref-103] Lindsey ARI, Bordenstein SR, Newton ILG, Rasgon JL (2016). Wolbachia pipientis should not be split into multiple species: a response to Ramírez-Puebla et al., “Species in Wolbachia? Proposal for the designation of ‘Candidatus Wolbachia bourtzisii’, ‘Candidatus Wolbachia onchocercicola’, ‘Candidatus Wolbachia blaxteri’, ‘Candidatus Wolbachia brugii’, ‘Candidatus Wolbachia taylori’, ‘Candidatus Wolbachia collembolicola’ and ‘Candidatus Wolbachia multihospitum’ for the different species within Wolbachia supergroups”. Systematic and Applied Microbiology.

[ref-104] Lindsey ARI, Rice DW, Bordenstein SR, Brooks AW, Bordenstein SR, Newton ILG (2018). Evolutionary genetics of cytoplasmic incompatibility genes cifA and cifB in prophage WO of Wolbachia. Genome Biology and Evolution.

[ref-105] Liu W, Xie Y, Ma J, Luo X, Nie P, Zuo Z, Lahrmann U, Zhao Q, Zheng Y, Zhao Y, Xue Y, Ren J (2015). IBS: an illustrator for the presentation and visualization of biological sequences. Bioinformatics.

[ref-106] Lo N, Casiraghi M, Salati E, Bazzocchi C, Bandi C (2002). How many Wolbachia supergroups exist?. Molecular Biology and Evolution.

[ref-107] Lomsadze A, Gemayel K, Tang S, Borodovsky M (2018). Modeling leaderless transcription and atypical genes results in more accurate gene prediction in prokaryotes. Genome Research.

[ref-108] Madhav M, Parry R, Morgan JAT, James P, Asgari S (2020). Wolbachia endosymbiont of the horn fly (haematobia irritans irritans): a supergroup a strain with multiple horizontally acquired cytoplasmic incompatibility genes. Applied and Environmental Microbiology.

[ref-109] Majerus TM, Majerus ME (2010). Discovery and identification of a male-killing agent in the Japanese ladybird Propylea japonica (Coleoptera: Coccinellidae). BMC Evolutionary Biology.

[ref-110] Makarova KS, Aravind L, Koonin EV (2000). A novel superfamily of predicted cysteine proteases from eukaryotes, viruses and Chlamydia pneumoniae. Trends in Biochemical Sciences.

[ref-111] Martijn J, Schulz F, Zaremba-Niedzwiedzka K, Viklund J, Stepanauskas R, Andersson SGE, Horn M, Guy L, Ettema TJG (2015). Single-cell genomics of a rare environmental alphaproteobacterium provides unique insights into Rickettsiaceae evolution. ISME Journal.

[ref-112] Martinez J, Klasson L, Welch JJ, Jiggins FM (2020). Life and death of selfish genes: comparative genomics reveals the dynamic evolution of cytoplasmic incompatibility. Molecular Biology and Evolution.

[ref-113] McCutcheon JP (2010). The bacterial essence of tiny symbiont genomes. Current Opinion in Microbiology.

[ref-114] McCutcheon JP (2016). From microbiology to cell biology: when an intracellular bacterium becomes part of its host cell. Current Opinion in Cell Biology.

[ref-115] McCutcheon JP, Boyd BM, Dale C (2019). The life of an insect endosymbiont from the cradle to the grave. Current Biology.

[ref-116] McNulty SN, Foster JM, Mitreva M, Hotopp JCD, Martin J, Fischer K, Wu B, Davis PJ, Kumar S, Brattig NW, Slatko BE, Weil GJ, Fischer PU (2010). Endosymbiont DNA in endobacteria-free filarial nematodes indicates ancient horizontal genetic transfer. PLOS ONE.

[ref-117] Meany MK, Conner WR, Richter SV, Bailey JA, Turelli M, Cooper BS (2019). Loss of cytoplasmic incompatibility and minimal fecundity effects explain relatively low Wolbachia frequencies in Drosophila mauritiana. Evolution.

[ref-118] Mediannikov O, Nguyen T-T, Bell-Sakyi L, Padmanabhan R, Fournier P-E, Raoult D (2014). High quality draft genome sequence and description of Occidentia massiliensis gen. nov., sp. nov., a new member of the family Rickettsiaceae. Standards in Genomic Sciences.

[ref-119] Merçot H, Charlat S (2004). Wolbachia infections in drosophila melanogaster and d. simulans: polymorphism and levels of cytoplasmic incompatibility. Genetica.

[ref-120] Metcalf JA, Jo M, Bordenstein SR, Jaenike J, Bordenstein SR (2014). Recent genome reduction of Wolbachia in Drosophila recens targets phage WO and narrows candidates for reproductive parasitism. PeerJ.

[ref-121] Misof B, Liu S, Meusemann K, Peters RS, Donath A, Mayer C, Frandsen PB, Ware J, Flouri T, Beutel RG, Niehuis O, Petersen M, Izquierdo-Carrasco F, Wappler T, Rust J, Aberer AJ, Aspock U, Aspock H, Bartel D, Blanke A, Berger S, Bohm A, Buckley TR, Calcott B, Chen J, Friedrich F, Fukui M, Fujita M, Greve C, Grobe P, Gu S, Huang Y, Jermiin LS, Kawahara AY, Krogmann L, Kubiak M, Lanfear R, Letsch H, Li Y, Li Z, Li J, Lu H, Machida R, Mashimo Y, Kapli P, McKenna DD, Meng G, Nakagaki Y, Navarrete-Heredia JL, Ott M, Ou Y, Pass G, Podsiadlowski L, Pohl H, von Reumont BM, Schutte K, Sekiya K, Shimizu S, Slipinski A, Stamatakis A, Song W, Su X, Szucsich NU, Tan M, Tan X, Tang M, Tang J, Timelthaler G, Tomizuka S, Trautwein M, Tong X, Uchifune T, Walzl MG, Wiegmann BM, Wilbrandt J, Wipfler B, Wong TKF, Wu Q, Wu G, Xie Y, Yang S, Yang Q, Yeates DK, Yoshizawa K, Zhang Q, Zhang R, Zhang W, Zhang Y, Zhao J, Zhou C, Zhou L, Ziesmann T, Zou S, Li Y, Xu X, Zhang Y, Yang H, Wang J, Wang J, Kjer KM, Zhou X (2014). Phylogenomics resolves the timing and pattern of insect evolution. Science.

[ref-122] Mockford LE, Krushelnycky DP (2008). New species and records of Liposcelis Motschulsky (Psocoptera: Liposcelididae) from Hawaii with first description of the male of Liposcelis bostrychophila Badonnel. Zootaxa.

[ref-123] Montagna M, Sassera D, Epis S, Bazzocchi C, Vannini C, Lo N, Sacchi L, Fukatsu T, Petroni G, Bandi C (2013). Candidatus Midichloriaceae fam. Nov. (Rickettsiales), an ecologically: widespread clade of intracellular alphaproteobacteria. Applied and Environmental Microbiology.

[ref-124] Moriyama M, Nikoh N, Hosokawa T, Fukatsu T (2015). Riboflavin provisioning underlies Wolbachia’s fitness contribution to its insect host. MBio.

[ref-125] Morrow JL, Schneider DI, Klasson L, Janitz C, Miller WJ, Riegler M (2020). Parallel sequencing of Wolbachia wCer2 from donor and novel hosts reveals multiple incompatibility factors and genome stability after host transfers. Genome Biology and Evolution.

[ref-126] Murray GGR, Weinert LA, Rhule EL, Welch JJ (2016). The phylogeny of rickettsia using different evolutionary signatures: how tree-like is bacterial evolution?. Systematic Biology.

[ref-127] Muñoz-Gómez SA, Hess S, Burger G, Franz Lang B, Susko E, Slamovits CH, Roger AJ (2019). An updated phylogeny of the alphaproteobacteria reveals that the parasitic rickettsiales and holosporales have independent origins. Elife.

[ref-128] Nakae S, Hijikata A, Tsuji T, Yonezawa K, Kouyama K-I, Mayanagi K, Ishino S, Ishino Y, Shirai T (2016). Structure of the EndoMS-DNA complex as mismatch restriction endonuclease. Structure.

[ref-129] Nakayama K, Kurokawa K, Fukuhara M, Urakami H, Yamamoto S, Yamazaki K, Ogura Y, Ooka T, Hayashi T (2010). Genome comparison and phylogenetic analysis of Orientia tsutsugamushi strains. DNA Research.

[ref-130] Nakayama K, Yamashita A, Kurokawa K, Morimoto T, Ogawa M, Fukuhara M, Urakami H, Ohnishi M, Uchiyama I, Ogura Y, Ooka T, Oshima K, Tamura A, Hattori M, Hayashi T (2008). The whole-genome sequencing of the obligate intracellular bacterium Orientia tsutsugamushi revealed massive gene amplification during reductive genome evolution. DNA Research.

[ref-131] Newton ILG, Rice DW (2020). The Jekyll and Hyde symbiont: could Wolbachia be a nutritional mutualist?. Journal of Bacteriology.

[ref-132] Nikoh N, Hosokawa T, Moriyama M, Oshima K, Hattori M, Fukatsu T (2014). Evolutionary origin of insect-Wolbachia nutritional mutualism. Proceedings of the National Academy of Sciences.

[ref-133] Nikoh N, McCutcheon JP, Kudo T, Miyagishima SY, Moran NA, Nakabachi A (2010). Bacterial genes in the aphid genome: absence of functional gene transfer from Buchnera to its host. PLOS Genetics.

[ref-134] Nogueras MM, Pons I, Ortuño A, Miret J, Pla J, Castellà J, Segura F (2013). Molecular detection of Rickettsia typhi in cats and fleas. PLOS ONE.

[ref-135] Nugnes F, Gebiola M, Monti MM, Gualtieri L, Giorgini M, Wang J, Bernardo U (2015). Genetic diversity of the invasive gall wasp Leptocybe invasa (Hymenoptera: Eulophidae) and of its rickettsia endosymbiont, and associated sex-ratio differences. PLOS ONE.

[ref-136] Oteo JA, Portillo A, Portero F, Zavala-Castro J, Venzal JM, Labruna MB (2014). ‘Candidatus Rickettsia asemboensis’ and Wolbachia spp. in Ctenocephalides felis and Pulex irritans fleas removed from dogs in Ecuador. Parasites & Vectors.

[ref-137] Penz T, Schmitz-Esser S, Kelly SE, Cass BN, Müller A, Woyke T, Malfatti SA, Hunter MS, Horn M (2012). Comparative genomics suggests an independent origin of cytoplasmic incompatibility in Cardinium hertigii. PLOS Genetics.

[ref-138] Perlmutter JI, Bordenstein SR, Unckless RL, LePage DP, Metcalf JA, Hill T, Martinez J, Jiggins FM, Bordenstein SR (2019). The phage gene wmk is a candidate for male killing by a bacterial endosymbiont. PLOS Pathogens.

[ref-139] Perotti MA, Clarke HK, Turner BD, Braig HR (2006). Rickettsia as obligate and mycetomic bacteria. Faseb Journal.

[ref-140] Pietri JE, DeBruhl H, Sullivan W (2016). The rich somatic life of Wolbachia. Microbiologyopen.

[ref-141] Poinsot D, Charlat S, Merçot H (2003). On the mechanism of *Wolbachia* -induced cytoplasmic incompatibility: confronting the models with the facts. BioEssays.

[ref-142] Pornwiroon W, Kearney MT, Husseneder C, Foil LD, Macaluso KR (2007). Comparative microbiota of Rickettsia felis-uninfected and -infected colonized cat fleas, Ctenocephalides felis. ISME Journal.

[ref-143] Prokopchuk G, Tashyreva D, Yabuki A, Horák A, Masařová P, Lukeš J (2019). Morphological, ultrastructural, motility and evolutionary characterization of two new Hemistasiidae Species. Protist.

[ref-144] Pruneda JN, Durkin CH, Geurink PP, Ovaa H, Santhanam B, Holden DW, Komander D (2016). The molecular basis for ubiquitin and ubiquitin-like specificities in bacterial effector proteases. Molecular Cell.

[ref-145] Ramírez-Puebla ST, Servín-Garcidueñas LE, Ormeño-Orrillo E, Vera-Ponce de León A, Rosenblueth M, Delaye L, Martínez J, Martínez-Romero E (2015). Species in Wolbachia? Proposal for the designation of ‘Candidatus Wolbachia bourtzisii’, ‘Candidatus Wolbachia onchocercicola’, ‘Candidatus Wolbachia blaxteri’, ‘Candidatus Wolbachia brugii’, ‘Candidatus Wolbachia taylori’, ‘Candidatus Wolbachia collembol. Systematic and Applied Microbiology.

[ref-146] Raychoudhury R, Baldo L, Oliveira DCSG, Werren JH (2009). Modes of acquisition of Wolbachia: horizontal transfer, hybrid introgression, and codivergence in the Nasonia species complex. Evolution.

[ref-147] Reif KE, Stout RW, Henry GC, Foil LD, Macaluso KR (2008). Prevalence and infection load dynamics of Rickettsia felis in actively feeding cat fleas. PLOS ONE.

[ref-148] Ren B, Kühn J, Meslet-Cladiere L, Briffotaux J, Norais C, Lavigne R, Flament D, Ladenstein R, Myllykallio H (2009). Structure and function of a novel endonuclease acting on branched DNA substrates. EMBO Journal.

[ref-149] Reveillaud J, Bordenstein SR, Cruaud C, Shaiber A, Esen ÖC, Weill M, Makoundou P, Lolans K, Watson AR, Rakotoarivony I, Bordenstein SR, Eren AM (2019). The Wolbachia mobilome in Culex pipiens includes a putative plasmid. Nature Communications.

[ref-150] Roberts LW, Rapmund G, Cadigan FC (1977). Sex ratios in rickettsia tsutsugamushi-infected and noninfected colonies of Leptotrombidium (Acari: trombiculidae). Journal of Medical Entomology.

[ref-151] Ros VID, Fleming VM, Feil EJ, Breeuwer JAJ (2009). How diverse is the genus Wolbachia? Multiple-gene sequencing reveals a putatively new Wolbachia supergroup recovered from spider mites (Acari: Tetranychidae). Applied and Environmental Microbiology.

[ref-152] Rust M (2017). The biology and ecology of cat fleas and advancements in their pest management: a review. Insects.

[ref-153] Ríhová J, Nováková E, Husník F, Hypša V (2017). Legionella becoming a mutualist: adaptive processes shaping the genome of symbiont in the Louse Polyplax serrata. Genome Biology and Evolution.

[ref-154] Satiaputra J, Shearwin KE, Booker GW, Polyak SW (2016). Mechanisms of biotin-regulated gene expression in microbes. Synthetic and Systems Biotechnology.

[ref-155] Schmidt TL, Barton NH, Rašić G, Turley AP, Montgomery BL, Iturbe-Ormaetxe I, Cook PE, Ryan PA, Ritchie SA, Hoffmann AA, O’Neill SL, Turelli M (2017). Local introduction and heterogeneous spatial spread of dengue-suppressing Wolbachia through an urban population of Aedes aegypti. PLOS Biology.

[ref-156] Schrallhammer M, Ferrantini F, Vannini C, Galati S, Schweikert M, Görtz H-D, Verni F, Petroni G (2013). Candidatus Megaira polyxenophila gen. nov., sp. nov.: considerations on evolutionary history, host range and shift of early divergent rickettsiae. PLOS ONE.

[ref-157] Schulz F, Martijn J, Wascher F, Lagkouvardos I, Kostanjšek R, Ettema TJG, Horn M (2016). A Rickettsiales symbiont of amoebae with ancient features. Environmental Microbiology.

[ref-158] Shapiro MM, Chakravartty V, Cronan JE (2012). Remarkable diversity in the enzymes catalyzing the last step in synthesis of the pimelate moiety of biotin. PLOS ONE.

[ref-159] Shi J, Cao X, Chen Y, Cronan JE, Guo Z (2016). An atypical α/β-hydrolase fold revealed in the crystal structure of pimeloyl-acyl carrier protein methyl esterase BioG from Haemophilus influenzae. Biochemistry.

[ref-160] Siozios S, Pilgrim J, Darby AC, Baylis M, Hurst GDD (2019). The draft genome of strain cCpun from biting midges confirms insect Cardinium are not a monophyletic group and reveals a novel gene family expansion in a symbiont. PeerJ.

[ref-161] Smith TA, Driscoll T, Gillespie JJ, Raghavan R (2015). A Coxiella-like endosymbiontis a potential vitamin source for the lone star tick. Genome Biology and Evolution.

[ref-162] Stamatakis A (2014). RAxML version 8: a tool for phylogenetic analysis and post-analysis of large phylogenies. Bioinformatics.

[ref-163] Sullivan JT, Brown SD, Yocum RR, Ronson CW (2001). The bio operon on the acquired symbiosis island of Mesorhizobium sp. strain R7A includes a novel gene involved in pimeloyl-CoA synthesis. Microbiology.

[ref-164] Sunyakumthorn P, Bourchookarn A, Pornwiroon W, David C, Barker SA, Macaluso KR (2008). Characterization and growth of polymorphic Rickettsia felis in a tick cell line. Applied and Environmental Microbiology.

[ref-165] Szokoli F, Castelli M, Sabaneyeva E, Schrallhammer M, Krenek S, Doak TG, Berendonk TU, Petroni G (2016). Disentangling the taxonomy of rickettsiales and description of two novel symbionts ("Candidatus Bealeia paramacronuclearis" and "Candidatus Fokinia cryptica") sharing the cytoplasm of the ciliate protist paramecium biaurelia. Applied and Environmental Microbiology.

[ref-166] Takahashi M, Urakami H, Yoshida Y, Furuya Y, Misumi H, Hori E, Kawamura A, Tanaka H (1997). Occurrence of high ratio of males after introduction of minocycline in a colony of Leptotrombidium fletcheri infected with Orientia tsutsugamushi. European Journal of Epidemiology.

[ref-167] Talavera G, Castresana J (2007). Improvement of phylogenies after removing divergent and ambiguously aligned blocks from protein sequence alignments. Systematic Biology.

[ref-168] Tashyreva D, Prokopchuk G, Votýpka J, Yabuki A, Horák A, Lukeš J (2018). Life cycle, ultrastructure, and phylogeny of new diplonemids and their endosymbiotic bacteria. MBio.

[ref-169] Tay ST (2013). *Wolbachia* endosymbionts, *Rickettsia felis* and *Bartonella* species, in *Ctenocephalides felis* fleas in a tropical region. Journal of Vector Ecology.

[ref-170] Traversa D (2013). Fleas infesting pets in the era of emerging extra-intestinal nematodes. Parasit Vectors.

[ref-171] Turelli M (1994). Evolution of incompatibility-inducing microbes and their hosts. Evolution.

[ref-172] Turelli M, Cooper BS, Richardson KM, Ginsberg PS, Peckenpaugh B, Antelope CX, Kim KJ, May MR, Abrieux A, Wilson DA, Bronski MJ, Moore BR, Gao JJ, Eisen MB, Chiu JC, Conner WR, Hoffmann AA (2018). Rapid global spread of wRi-like Wolbachia across multiple drosophila. Current Biology.

[ref-173] Turelli M, Hoffmann AA (1991). Rapid spread of an inherited incompatibility factor in California Drosophila. Nature.

[ref-174] Vasconcelos EJR, Billeter SA, Jett LA, Meinersmann RJ, Barr MC, Diniz PPVP, Oakley BB (2018). Assessing cat flea microbiomes in Northern and Southern California by 16S rRNA next-generation sequencing. Vector-Borne and Zoonotic Diseases.

[ref-175] Von der Schulenburg JH, Habig M, Sloggett JJ, Webberley KM, Bertrand D, Hurst GD, Majerus ME (2001). Incidence of male-killing Rickettsia spp. (alpha-proteobacteria) in the ten-spot ladybird beetle Adalia decempunctata L. (Coleoptera: Coccinellidae). Applied and Environmental Microbiology.

[ref-176] Werren JH, Baldo L, Clark ME (2008). Wolbachia: master manipulators of invertebrate biology. Nature Reviews Microbiology.

[ref-177] Werren JH, Hurst GD, Zhang W, Breeuwer JA, Stouthamer R, Majerus ME (1994). Rickettsial relative associated with male killing in the ladybird beetle (Adalia bipunctata). Journal of Bacteriology.

[ref-178] Werren JH, Richards S, Desjardins CA, Niehuis O, Gadau J, Colbourne JK, Desplan C, Elsik CG, Grimmelikhuijzen CJP, Kitts P, Lynch JA, Murphy T, Oliveira DCSG, Smith CD, Zande Lvd, Worley KC, Zdobnov EM, Aerts M, Albert S, Anaya VH, Anzola JM, Barchuk AR, Behura SK, Bera AN, Berenbaum MR, Bertossa RC, Bitondi MMG, Bordenstein SR, Bork P, Bornberg-Bauer E, Brunain M, Cazzamali G, Chaboub L, Chacko J, Chavez D, Childers CP, Choi J-H, Clark ME, Claudianos C, Clinton RA, Cree AG, Cristino AS, Dang PM, Darby AC, de Graaf DC, Devreese B, Dinh HH, Edwards R, Elango N, Elhaik E, Ermolaeva O, Evans JD, Foret S, Fowler GR, Gerlach D, Gibson JD, Gilbert DG, Graur D, Grunder S, Hagen DE, Han Y, Hauser F, Hultmark D, Hunter HC, Hurst GDD, Jhangian SN, Jiang H, Johnson RM, Jones AK, Junier T, Kadowaki T, Kamping A, Kapustin Y, Kechavarzi B, Kim J, Kim J, Kiryutin B, Koevoets T, Kovar CL, Kriventseva EV, Kucharski R, Lee H, Lee SL, Lees K, Lewis LR, Loehlin DW, Logsdon JM, Lopez JA, Lozado RJ, Maglott D, Maleszka R, Mayampurath A, Mazur DJ, McClure MA, Moore AD, Morgan MB, Muller J, Munoz-Torres MC, Muzny DM, Nazareth LV, Neupert S, Nguyen NB, Nunes FMF, Oakeshott JG, Okwuonu GO, Pannebakker BA, Pejaver VR, Peng Z, Pratt SC, Predel R, Pu L-L, Ranson H, Raychoudhury R, Rechtsteiner A, Reid JG, Riddle M, Romero-Severson J, Rosenberg M, Sackton TB, Sattelle DB, Schluns H, Schmitt T, Schneider M, Schuler A, Schurko AM, Shuker DM, Simoes ZLP, Sinha S, Smith Z, Souvorov A, Springauf A, Stafflinger E, Stage DE, Stanke M, Tanaka Y, Telschow A, Trent C, Vattathil S, Viljakainen L, Wanner KW, Waterhouse RM, Whitfield JB, Wilkes TE, Williamson M, Willis JH, Wolschin F, Wyder S, Yamada T, Yi SV, Zecher CN, Zhang L, Gibbs RA (2010). Functional and evolutionary insights from the genomes of three parasitoid nasonia species. Science.

[ref-179] Wilson ACC, Duncan RP (2015). Signatures of host/symbiont genome coevolution in insect nutritional endosymbioses. Proceedings of the National Academy of Sciences.

[ref-180] Yang Q, Kučerová Z, Perlman SJ, Opit GP, Mockford EL, Behar A, Robinson WE, Stejskal V, Li Z, Shao R (2015). Morphological and molecular characterization of a sexually reproducing colony of the booklouse Liposcelis bostrychophila (Psocodea: Liposcelididae) found in Arizona. Scientific Reports.

[ref-181] Yurchenko T, Ševčíková T, Přibyl P, El Karkouri K, Klimeš V, Amaral R, Zbránková V, Kim E, Raoult D, Santos LMA, Eliáš M (2018). A gene transfer event suggests a long-term partnership between eustigmatophyte algae and a novel lineage of endosymbiotic bacteria. ISME Journal.

[ref-182] Zeng Z, Fu Y, Guo D, Wu Y, Ajayi OE, Wu Q (2018). Bacterial endosymbiont Cardinium cSfur genome sequence provides insights for understanding the symbiotic relationship in Sogatella furcifera host. BMC Genomics.

[ref-183] Zeng Q, Yang Q, Jia J, Bi H (2020). A moraxella virulence factor catalyzes an essential esterase reaction of biotin biosynthesis. Frontiers in Microbiology.

[ref-184] Zhang D, De Souza RF, Anantharaman V, Iyer LM, Aravind L (2012). Polymorphic toxin systems: comprehensive characterization of trafficking modes, processing, mechanisms of action, immunity and ecology using comparative genomics. Biology Direct.

[ref-185] Zug R, Hammerstein P (2015). Bad guys turned nice? A critical assessment of Wolbachia mutualisms in arthropod hosts. Biological Reviews.

