# Supplementary figures and images for: Evolution of Wolbachia mutualism and reproductive parasitism: insight from two novel strains that co-infect cat fleas
Source: PeerJ. 2020 Dec 17;8:e10646. doi: 10.7717/peerj.10646 (PMC7750005; doi:10.7717/peerj.10646)

A

Fig. S1

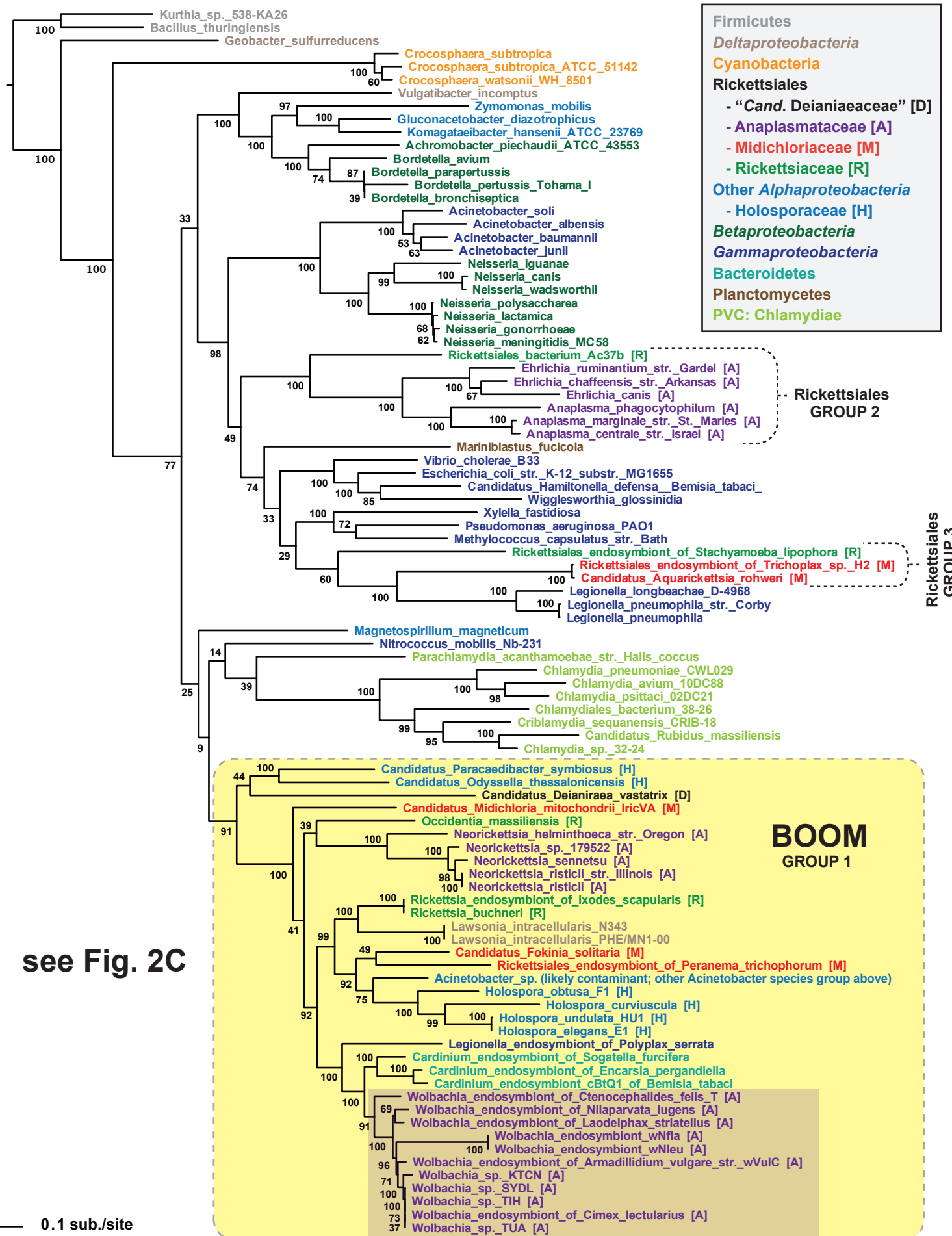

B

Fig. S1

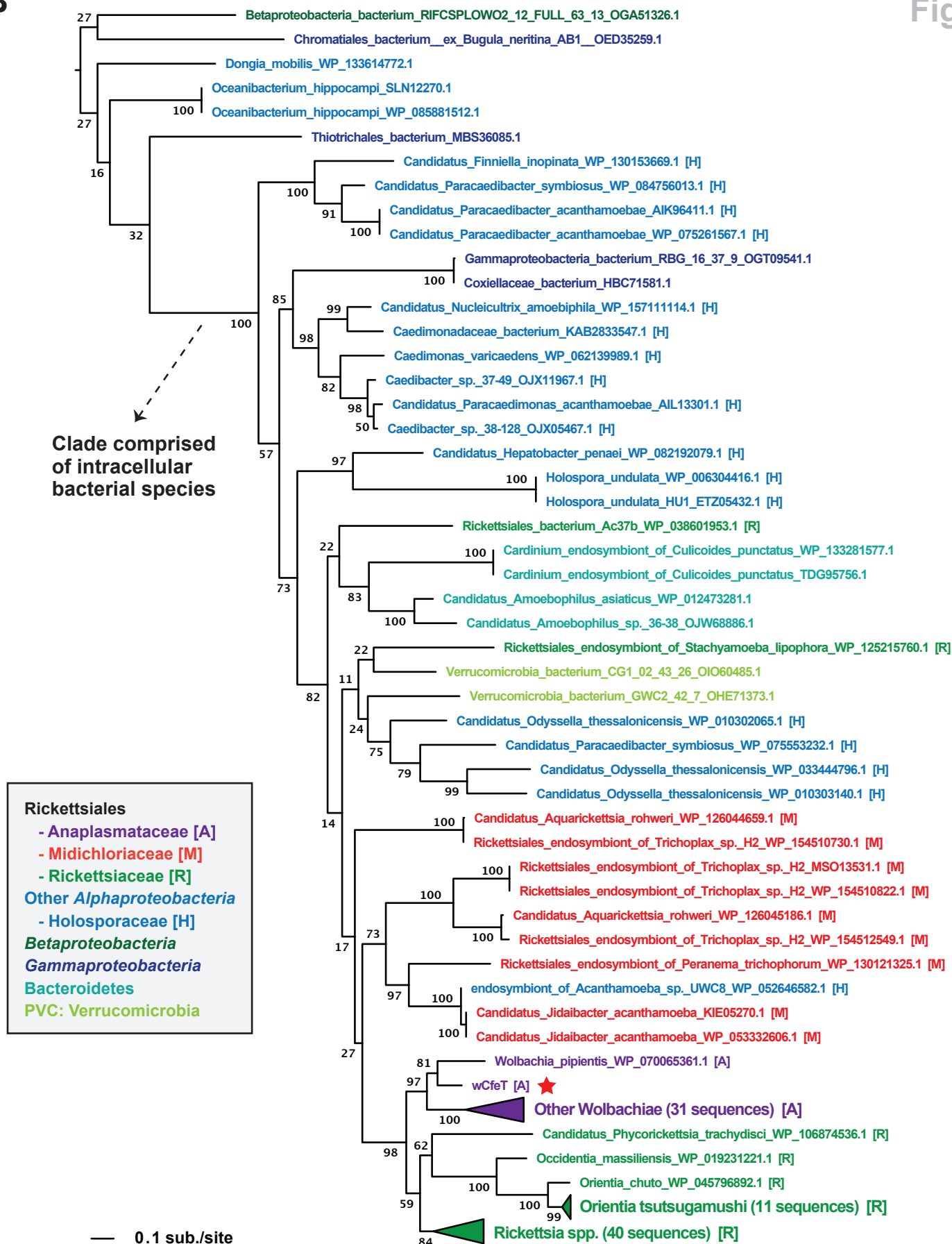

Supplement: Supplemental Information 1 — (A) Complete phylogeny estimation of BOOM and other bio gene sets from diverse bacteria. Tree was estimated from the concatenation of six bio enzymes (BioC, BioH, BioF, BioA, BioD, and BioB) or subsets in certain cases (see Table S2 for all sequence information and Materials and Methods for details on dataset processing and tree estimation). Branch support was assessed with 1,000 pseudo-replications. Final ML optimization likelihood was -127140.955066. In taxon color scheme, [R] denotes Rickettsiales, with Holosporaceae [H] as a revised family of Rhodospirillales (101). The families of Rickettsiales are similarly noted: Anaplasmataceae [A], Midichloriaceae [M], Rickettsiaceae [R], and “Candidatus Deianiaeaceae” [D], with the latter considered provisional (91). (B) Estimated phylogeny of EamA transporters. See Materials and Methods for details on dataset processing and tree estimation. Branch support was assessed with 1,000 pseudo-replications. Final ML optimization likelihood was -23058.797227. Taxon color scheme as described in panel A. [file peerj-08-10646-s001.pdf]
