# Supplementary material for: Evolution of Wolbachia mutualism and reproductive parasitism: insight from two novel strains that co-infect cat fleas
Source: PeerJ. 2020 Dec 17;8:e10646. doi: 10.7717/peerj.10646 (PMC7750005; doi:10.7717/peerj.10646)
Supplement: Supplemental Information 2 — Schema shows the comparison of the wCfeT and WOVitA1 prophage genomes, with gene descriptions following prior studies (26, 30, 148, 183). wCfeT genes within the typical Eukaryotic Association Module (EAM) were used as queries in Blastp searches against the NCBI nr protein database, with summary statistics provided for top subjects (dashed box). [file peerj-08-10646-s002.pdf]

*w*CfeT proteins		Top Blastp hit							
Protein	Size	Accession	Size	Annotation	Taxon	Max, tot.	Cov.	E value	%ID
WP_168464803	309	APR98706	311	Patatin-like phospholipase	*w*Fol	514, 514	100%	0.0	78.14%
WP_168464804	72	AZU37351	74	ParD-like family antidote	*w*Bta	107, 107	97%	7e-29	72.86%
WP_168464805	96	OAM00647	92	RelE/ParE family toxin	*w*DacA	154, 154	95%	5e-47	80.43%
---------------------	44	----------------	----	--	----------	---------------	--------	--------	-----------
WP_168464806	262	APR98707	257	PHA03095 (Ank repeats)	*w*Fol	321, 321	98%	1e-107	60.31%
WP_168464807	82	APR99034	86	XRE family transcriptional regulator	*w*Fol	111, 111	97%	5e-30	68.75%
WP_168464808	648	APR98618	651	DNA ligase (NAD(+)) LigA	*w*Fol	1007, 1007	99%	0.0	73.92%
WP_168464809	79	APR97852	96	XRE family transcriptional regulator	*w*Fol	139, 139	98%	4e-41	89.74%
WP_168464414	545	WP_143688845	493	Group II intron RevTranscriptase/maturase	*w*Str	992, 992	89%	0.0	99.59%
WP_168464810	310	APR98606	312	Recombination-promoting nuclease/put. tnp	*w*Fol	506, 506	100%	3e-179	79.35%
WP_168464811	301	APR98615	302	Hypothetical protein (put. rhoptry protein)	*w*Fol	421, 421	100%	9e-146	74.17%
WP_168464812	226	APR98945	217	DNA repair protein RadC	*w*Fol	367, 367	96%	7e-127	84.86%

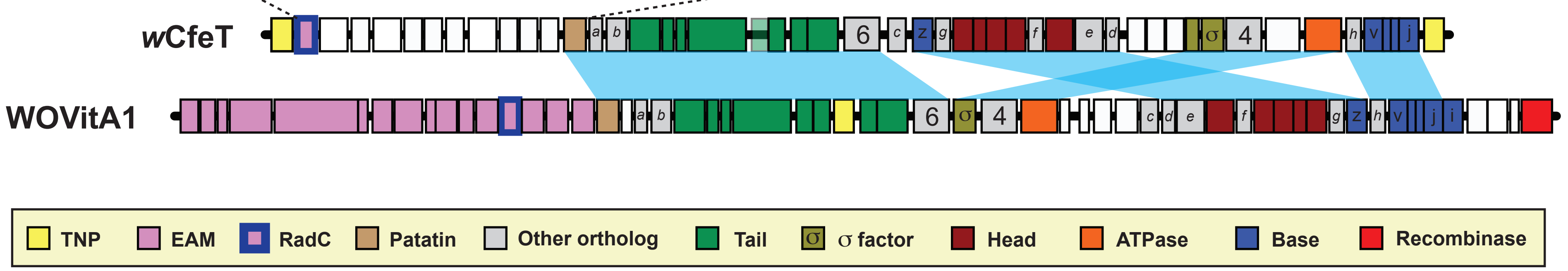

Fig. S2
