# Supplementary material for: Evolution of Wolbachia mutualism and reproductive parasitism: insight from two novel strains that co-infect cat fleas
Source: PeerJ. 2020 Dec 17;8:e10646. doi: 10.7717/peerj.10646 (PMC7750005; doi:10.7717/peerj.10646)
Supplement: Supplemental Information 3 — Rickettsiales taxa with at least 5 of 6 biotin synthesis genes (n=28) were analyzed for co-linearity of their biotin genes. Ortholog groups (n=2,527) were constructed from these genomes as described for Fig. 1 (see Materials and Methods) and OGs containing biotin synthesis genes identified. Genome locations were retrieved from NCBI in gene file format (gff) and used to order the orthologs in each genome. For unclosed genomes, all contigs were included in OG construction; only a single genome (Candidatus Aquarickettsia rohweri) contained biotin synthesis genes on multiple contigs. Biotin genes are colored according to the ideal BOOM shown at the top. Black boxes indicate stretches of unrelated genes. Taxa are grouped into 3 classes according to the synteny of biotin genes in their genomes: Class I taxa demonstrate completely conserved gene order; Class II taxa contain only a single gene out of order (Class IIA: BioA; Class IIB: BioB); Class III taxa exhibit little or no conservation of gene order. All blocks are anchored on BioB for ease of comparison, except Class IIB which are anchored on BioF instead. [file peerj-08-10646-s003.pdf]

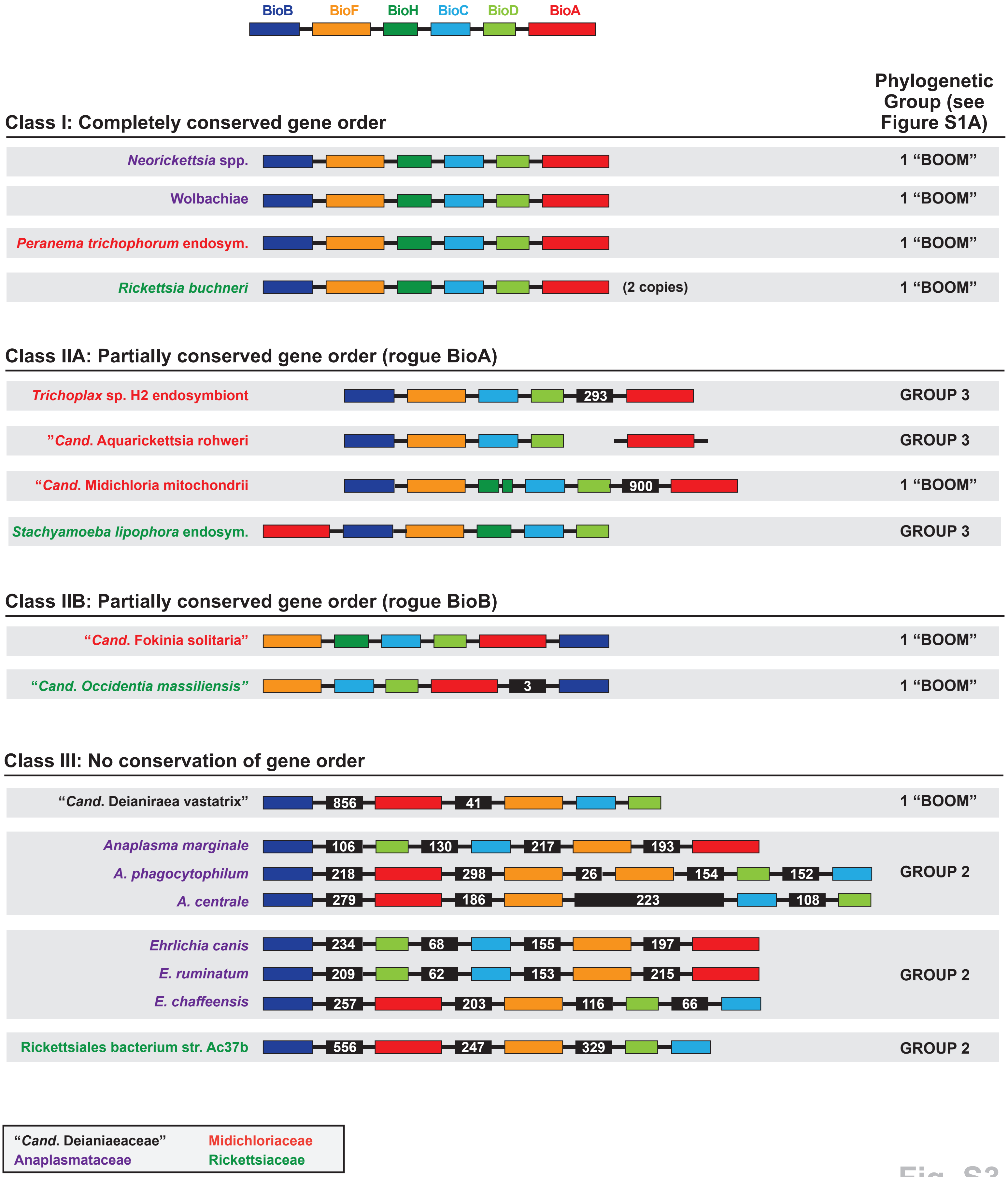

BioB
BioF
BioH
BioC
BioD
BioA
Phylogenetic Group (see Figure S1A)
Class I: Completely conserved gene order
Neorickettsia spp.
1 “BOOM”
Wolbachiae
1 “BOOM”
Peranema trichophorum endosym.
1 “BOOM”
Rickettsia buchneri
(2 copies)
1 “BOOM”
Class IIA: Partially conserved gene order (rogue BioA)
Trichoplax sp. H2 endosymbiont
293
GROUP 3
”Cand. Aquarickettsia rohweri
GROUP 3
“Cand. Midichloria mitochondrii
900
1 “BOOM”
Stachyamoeba lipophora endosym.
GROUP 3
Class IIB: Partially conserved gene order (rogue BioB)
“Cand. Fokinia solitaria”
1 “BOOM”
“Cand. Occidentia massiliensis”
3
1 “BOOM”
Class III: No conservation of gene order
“Cand. Deianiraea vastatrix”
856
41
1 “BOOM”
Anaplasma marginale
106
130
217
193
A. phagocytophilum
218
298
26
154
152
A. centrale
279
186
223
108
GROUP 2
Ehrlichia canis
234
68
155
197
E. ruminatum
209
62
153
215
E. chaffeensis
257
203
116
66
GROUP 2
Rickettsiales bacterium str. Ac37b
556
247
329
GROUP 2
“Cand. Deianiaeaceae”
Midichloriaceae
Anaplasmataceae
Rickettsiaceae

Fig. S3
