# Supplementary material for: Evolution of Wolbachia mutualism and reproductive parasitism: insight from two novel strains that co-infect cat fleas
Source: PeerJ. 2020 Dec 17;8:e10646. doi: 10.7717/peerj.10646 (PMC7750005; doi:10.7717/peerj.10646)
Supplement: Supplemental Information 4 — (A) Alignment of Ehrlichia chaffeensis and Anaplasma centrale BioU proteins with orthologs from other Rickettsiales species. BioU orthologs were retrieved from Blastp searches against the NCBI Rickettsiales databse using E. chaffeensis str. Sapulpa BioU (EAM85518.1) as a query. The catalytic residues (Ser–Asp–His) characteristic of methyl esterases are highlighted yellow (184). Sequences were aligned with MUSCLE v3.8.31 (169) using default parameters. NCBI protein accession numbers are listed in panel C. (B) Percent identity matrix calculated from the alignment shown in panel A. (C) Estimated phylogeny of proteins shown in panel A. Two alphaproteobacterial outgroups and two hymenopteran mitochondrial methyl esterases are included. Phylogeny was estimated using RAxML v8.2.4 (171) under the gamma model of rate heterogeneity and estimation of the proportion of invariant sites. Branch support was assessed with 1,000 pseudo-replications. Final ML optimization likelihood was -5448.767799. (D) Analysis of eight rickettsial genomes that contain biotin synthesis genes except for the BioH methyl esterase. Blastp searches against the rickettsial genomes were conducted using the following queries, all of which have been shown to orthogonally substitute for BioH: BioV of Helicobacter sp. L8b (TSA87024.1); BioG of Haemophilus influenzae str. CGSHiCZ412602 (AIB45959.1); BtsA of Moraxella catarrhalis str. CCUG 353 (KZR94829.1); BioJ of Francisella marina str. E103-15 (QEO56965.1); BioZ of Mesorhizobium japonicum str. R7A (AAG47795.1); EstN1 of Nitrososphaera viennensis str. EN76 (AIC14436.1); BioK of Prochlorococcus marinus str. SS51 (KGG31655.1). [file peerj-08-10646-s004.pdf]

A

```
U ┤ Ehrlichia chaffeensis             ------MSDHYK--LVLSNPQGLHITYRQLLGN----KASIIFFGGFNSNMQGTKATALY    48
  │ Anaplasma centrale                MLRQSCVGAVVGEEKRLELGNSSYISYMQTTVQS---PVSVVFFGGFMSDMHGTKAQHLF    57
Rickettsiales bacterium Ac37b         ------MNIAFN-VQKLLLSNNNYIAYSKINSKTQNELPGIIFFSGFNSNMQGTKARNLT    53
Neorickettsia helminthoeca            ------MQTKYL------DTSHGRIAYQTFDNNP---EVGVLFMTGLASDMSGRKSERLR    45
Rickettsia buchneri                   ------MHKLYN------KTQDKFIVYDNYRIINTN-IPSVIFLHGLMSSMKSTKAIYLI    47
wCle                                  -------MDYCK----LFGESGKYIAYRKLQGR----RTSIVFFGGFASNMNGTKATAIY    45
● wPip_Pel                            -------MNYCK----LLDGSNGHIAYRKLQGK----KASIVFFSGFASNMDGTKATAVY    45
● Neoehrlichia lotoris                ------MTDYSPSFLVLDHNNNHKIAYRQLKKNSN--LPSILLLGGFGSNMYGEKATALY    52
● Rickettsia typhi                    ------MHKLYN------KTQDKFIVYDNYRIINTN-IPSVIFLHGLMSSMQSTKAIYLI    47
● Orientia tsutsugamushi Boryong      ------MHKIYT------LDDGRFIAYRQHKSQKNS-LINIIFLHGMMSNMSGKKSSYLY    47
                                                          .   * *            .:::: *: *.* . *:  :

                                                                                       Ser
U ┤ Ehrlichia chaffeensis             DYCKSHNLGLILFDYLGHGQSDGQFTDYNISDWYKNCIEIITQLTPTNRPKIIIGSSMGA   108
  │ Anaplasma centrale                EYCKSHGVHCTVFDYFGHGSSSGEFQECTISDWYASCVSVVESL--TSAPLVIVGSSMGG   115
Rickettsiales bacterium Ac37b         EYCQNNNYNFIKFDYLGHGLSSGIFHECTIGIWLENCLSIIDNL--TTDKHIFIGSSMGG   111
Neorickettsia helminthoeca            SFCENNQVAFTRFDYFGHGRSEGSFLHGNISKWTENALEVLERV--TTGKQILVGSSMSG   103
Rickettsia buchneri                   DYCKKNNYNFIVFDNFGHGNASGQFEDQTISDWLEGVSLILDKL--IDKEAILVGSSMGG   105
wCle                                  KFCQENDVALVLFDYFGHGHSSGDFTDYTISDWQKNCTRVINEL--TSSKQIIIGSSMGG   103
● wPip_Pel                            KFCQENDIALVLFDYFGHGNSSGDFADYTISDWQKNCAKVISEL--TSNKQIIIGSSMGG   103
● Neoehrlichia lotoris                NYCNKHNLNLTVFDYLGHGHSSGNFTDYTIGDWYKNCISVIESL--TNGPQIIIGSSMGG   110
● Rickettsia typhi                    DYCKKNNYNFIVFDNFGHGNAYGQFEDQTISDWLEGVALILDKL--IETEAILIGSSMGG   105
● Orientia tsutsugamushi Boryong      QLCQEEDLNFLAFDNYGHGNSSGRFIDQTIESWFDATRAIMYHTS-NNFKNIIVGSSLGG   106
                                      . *:.        **   *** : * *    .*   *        ::         :::***:..

U ┤ Ehrlichia chaffeensis             WLMLLVAISHQDKVSHLISLAGAPDFTESLIFQKLNTQQKDELYKYGQITL--SQNSNNM   166
  │ Anaplasma centrale                WLMLLTALSHGRRVRGLVGMAPAPDFTESL---DLSESQRAEMMRTGKTIK--NTDNC--   168
Rickettsiales bacterium Ac37b         WLALLASILRPEKVAGIICIAAAPDFTENLIWNTLSLEEKNKLQTQGIIKL--SSNYCEG   169
Neorickettsia helminthoeca            WMMFKIAEKHPEKVKGLVGIAAAPDFTED-FLEGLTHETKQALEKNGYFTF--TRNRDE-   159
Rickettsia buchneri                   WLALLAALRFPDKIKGLVCVAPAPDFTEN-IWQNISLNDQNKMQKEGILEV--SGKNCEH   162
wCle                                  WLMLLTALQIPERIAALIGVSSAPDFTEDLIFKQLSGKQKEELDSKGVVDF--TSGRC--   159
● wPip_Pel                            WLMLLTALQFPEKIAALIGISSAPDFTEDLIFKQLSGKQKEELGSKGVIDF--TSEHC--   159
● Neoehrlichia lotoris                WLMLLIAQSYPHKVISLLGLAPAPDFTENLIFNKLTQEQKDCLHTNNQIIF--TFNKYED   168
● Rickettsia typhi                    WLALLAALRFPDKIKCLICVAPALDFTEN-IWQNISLNDQNKMKKEGIIEV--SSENCQH   162
● Orientia tsutsugamushi Boryong      WLAMLAAIKNEIEISGVVALAPAIDFTETLIWNKLTEKNKNSMIHTGYIELGGTGNTCNN   166
                                      *: :  :       :  :: :: * ****       :. . .  :    .       .

                                                                      Asp
U ┤ Ehrlichia chaffeensis             YSYVITRNLIEDGRKHLLLNQESINITCPITLIHGMNDDTVPYQTSITVAEKIKSDNVNL   226
  │ Anaplasma centrale                -SYVITKKLIDDGKAHLLMNKREIAVECPMVLIHGMDDTVVPYQVSLEIAGKVKSGDVRV   227
Rickettsiales bacterium Ac37b         -EYEISLKLIEEAREHLILNK-PLDIKCPIYLLHGMADKDVPYNFSLDLVNSISSQDITV   227
Neorickettsia helminthoeca            -KLVITKTLLDDGKKNLILTQ-RIKVPCPVVLLHGLADDIVSYRKSIELAELIESSPVEV   217
Rickettsia buchneri                   -KYPISYKLIEDAKKHLLLTKKQIDINTPLHIIHGMLDEDVPYNVSVKLLEKITSKQIVM   221
wCle                                  -AYKITKNLIEDGRKKLLLNKETIDINSPVRLLHSINDKDVPYQTSLNLAERVKSTDVEV   218
● wPip_Pel                            -AYKITKNLIEDGRKNLLLNREAIDINCPVRLLHSINDKDVPYQTSLNLAEKIKSTDVEV   218
● Neoehrlichia lotoris                RSYDITDNLIKDGRKHLLLNNDNININCPVILIHSMSDLVVPYSTSIHVAEKITSTNVNL   228
● Rickettsia typhi                    -KYPISYKLIEDAKKHLLLRKQQIEINIPVHIIHGMLDKNVPYNVSVKLLEKITSKQIVM   221
● Orientia tsutsugamushi Boryong      -KYHISYNLICNARKYLLLNKPTINIQCPIAIIHGMQDQEVPYQGSIDLINKVQTHYSTL   225
                                          *: .*: :..  *:: .  : :  *: ::*.: *  *.*  *: :   : :   :

                    His
U ┤ Ehrlichia chaffeensis             HLIKSANHNLSDDTSLNIIFKYIKEAVEQSIQVK   260
  │ Anaplasma centrale                HLSKSGTHRLTDEHSLGLMLESVKGLMRPSVPV-   260
Rickettsiales bacterium Ac37b         KLVKDAGHGMSTKVNLYLLYNTINELITKITNK-   260
Neorickettsia helminthoeca            RLIKGADHSMSDPTSITVLTDTVRALI-------   244
Rickettsia buchneri                   KLIKDGHHNLSREEDLKVMTNSLEEVISLSNIK-   254
wCle                                  HLTKSAEHNMSDSHSLKILFQAIREFLPTV----   248
● wPip_Pel                            HLIKSAEHNMSDNHSLKILFKTIREFLPGEIYN-   251
● Neoehrlichia lotoris                HLIKSGNHYLRDEHSLNVTFSAIQSLLAQC----   258
● Rickettsia typhi                    KLIKDGNHNLSRKEDLKVIANSLEEMISNIK---   252
● Orientia tsutsugamushi Boryong      KLLKYADHFLSDSVSLSHISYAIKEIINARLV--   257
                                      .* * . * :    .:      :   :
```

U = characterized BioU

▭ = taxa with BioH (boxed: Neorickettsia helminthoeca, Rickettsia buchneri, wCle)

● = no biotin synthesis

Fig. S4

Fig. S4

B

	Otsu	Nhel	Acen	Rtyp	Rbuc	R37b	wCle	wPip_Pel	Nlot	Echa
● *Orientia tsutsugamushi* Boryong (Otsu)	100.00									
Neorickettsia helminthoeca (Nhel)	31.56	100.00								
U *Anaplasma centrale* (Acen)	34.01	37.08	100.00							
● *Rickettsia typhi* (Rtyp)	40.48	31.97	35.92	100.00						
Rickettsia buchneri (Rbuc)	41.50	34.43	38.87	88.49	100.00					
Rickettsiales bacterium Ac37b (R37b)	41.11	37.30	36.90	46.22	45.85	100.00				
*w*Cle	38.21	40.25	44.90	37.14	36.73	44.13	100.00			
● *w*Pip_Pel	37.10	43.15	43.15	39.43	37.50	46.80	84.27	100.00		
● *Neoehrlichia lotoris* (Nlot)	40.24	38.93	45.82	37.60	37.60	41.57	52.02	52.82	100.00	
U *Ehrlichia chaffeensis* (Echa)	41.67	37.45	45.02	41.37	39.44	41.96	52.82	54.98	60.24	100.00

C

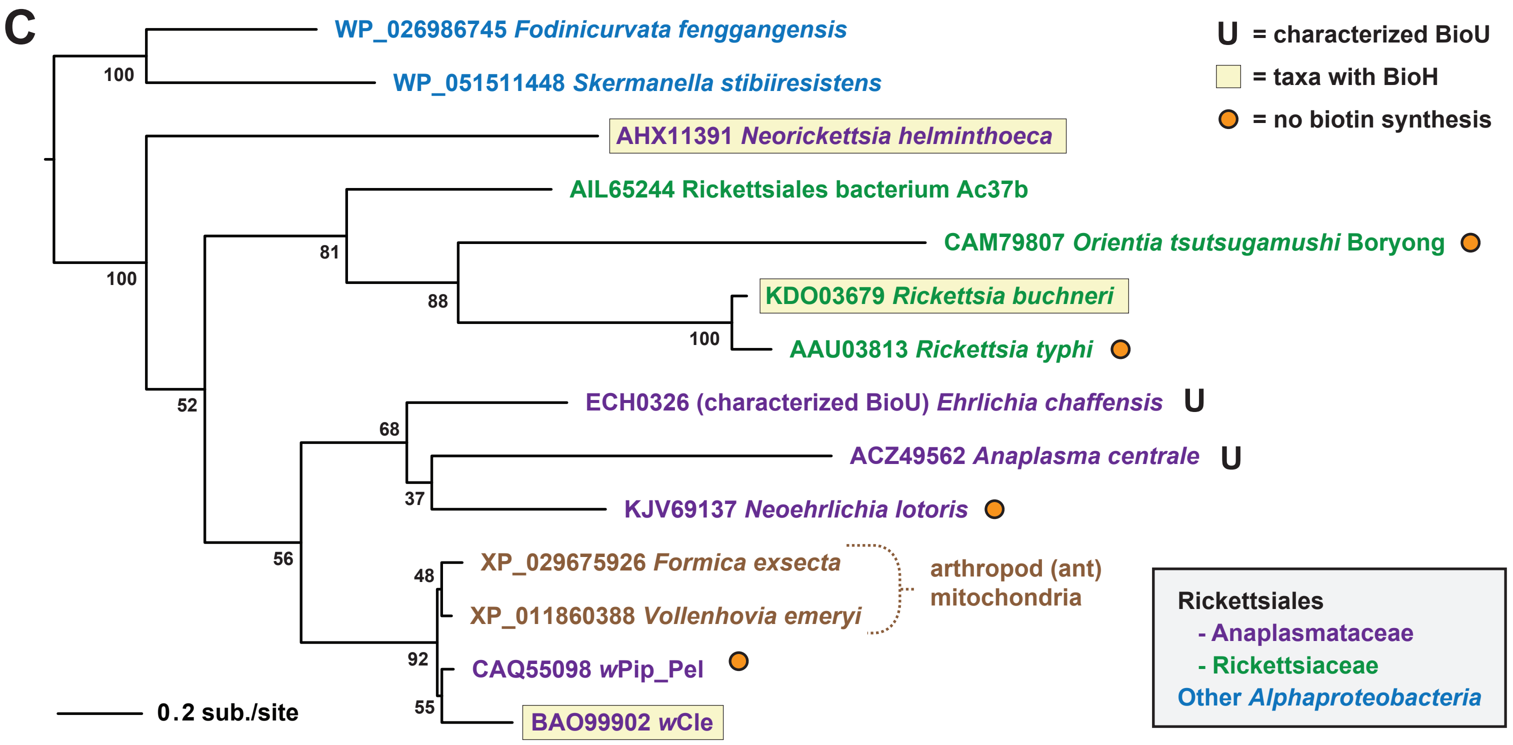

D

NCBI taxid	Taxon	Blastp results (%ID) BioV	BioG	BtsA	BioJ	BioZ *	EstN1	BioK	BioU
768	Anaplasma	NSS	NSS	NSS	NSS	33	~27; (2)	NSS	Present
943	Ehrlichia	NSS	NSS	NSS	NSS	33	~27; (2)	NSS	Present
2021221	Rickettsiales endosymbiont of Trichoplax sp. H2	NSS	NSS	NSS	NSS	33	NSS	NSS	NSS
2602574	Ca. Aquarickettsia rohweri	NSS	NSS	NSS	NSS	33	NSS	NSS	NSS
1528098	Rickettsiales bacterium Ac37b	NSS	NSS	NSS	NSS	35	31; AIL65750	NSS	Present
752179	Occidentia massiliensis	NSS	NSS	NSS	NSS	35	NSS	NSS	NSS
2163644	Ca. Deianiraea vastatrix	NSS	NSS	37; (1)	NSS	30	NSS	NSS	NSS

(1) QED23496 (5-methyltetrahydropteroyltriglutamate--homocysteine S-methyltransferase); only 12% query coverage

* % similarity to FabH; BioZ is similar to FabH but was able to complement *E. coli ΔbioH* mutants (PMID: 11320134).

(2) similarity to a cohort of MhpC-like Abhydrolase 5 proteins (unrelated to BioU; *E. chaffeensis* Arkansas protein is ECH_0221).
